# TNF/TNFR1 axis restrains inflammation-suppressive myeloid subclusters to drive acute pancreatitis

**DOI:** 10.1038/s41419-026-09208-9

**Published:** 2026-08-26

**Authors:** Pan Zheng, Xueyang Li, Yuman Ye, Yaoyu Zou, Maobin Kuang, Jingwen Gao, Dongni Fu, Jianhua Wan, Cong He, Nonghua Lu, Xiaoli Xi, Nianshuang Li, Yin Zhu

**Affiliations:** 1 Department of Gastroenterology, Jiangxi Provincial Key Laboratory of Digestive Diseases, Jiangxi Clinical Research Center for Gastroenterology, Digestive Disease Hospital, The First Affiliated Hospital, Jiangxi Medical College, Nanchang University, Nanchang, Jiangxi China; 2 Postdoctoral Innovation Practice Base, The First Affiliated Hospital, Jiangxi Medical College, Nanchang University, Nanchang, Jiangxi China; 3 Huankui Academy, Jiangxi Medical College, Nanchang University, Nanchang, Jiangxi China

**Keywords:** Acute inflammation, Acute pancreatitis

## Abstract

Acute pancreatitis (AP) is a pancreatic inflammatory disorder that can progress to fatal systemic inflammation and multiorgan failure. Although tumor necrosis factor (TNF) signaling is a key regulator of inflammation, its contribution to AP progression and its therapeutic potential remain incompletely understood. We induced AP in wild-type and *Tnfr1*^–/–^ mice using caerulein, pancreatic duct ligation, or L-arginine. Single-cell RNA sequencing (scRNA-seq) and bulk RNA sequencing were used to characterize pancreatic immune-cell profiles, and pomalidomide, infliximab, and necrostatin-1 were evaluated as pharmacological interventions targeting the TNF/TNFR1 pathway. Analysis of public scRNA-seq datasets revealed activation of the TNF/TNFR1 axis across three AP models, accompanied by increased TNF-α and TNFR1 expression. *Tnfr1* deletion markedly attenuated pancreatic injury and inflammatory-cell infiltration. scRNA-seq of pancreatic tissues further revealed substantial remodeling of the immune landscape in *Tnfr1*^–/–^ mice, including reductions in myeloid cells and neutrophils. Within the Neu_C3 neutrophil subset, NF-κB and TNF signaling and neutrophil extracellular trap formation were suppressed. Integrated analysis with bulk RNA-seq showed reduced expression of *Marcksl1*, *Ifit3*, and *Oas3* in the Neu_C3 cluster, with further reductions following TNFR1 ablation. *Tnfr1* deletion also reduced M1 macrophage polarization and expanded an Igkc-high myeloid subcluster characterized by suppressed chemokine signaling. Among the three compounds tested, pomalidomide showed the strongest preventive and therapeutic effects across the experimental AP models. Together, these findings indicate that the TNF/TNFR1 axis promotes AP by shaping inflammatory myeloid-cell states. Targeting this pathway, particularly with pomalidomide, may provide a therapeutic strategy for AP by remodeling immune-cell populations and suppressing inflammation.

**Figure:**
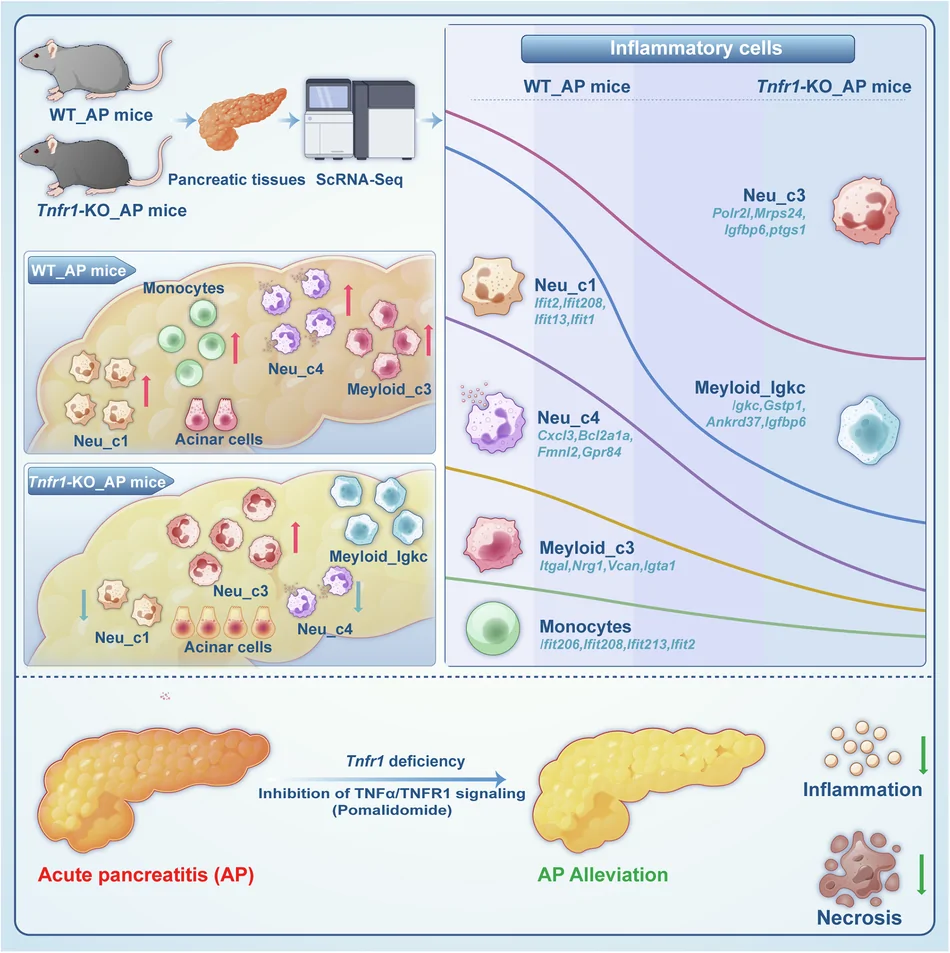

## Introduction

Acute pancreatitis (AP) is a critical abdominal emergency commonly associated with etiological factors including alcohol consumption, biliary obstruction, and hyperlipidemia [1]. While mild AP cases typically exhibit self-limiting characteristics with spontaneous resolution within days, a subset progresses to severe acute pancreatitis (SAP) [2]. AP is initiated by etiological agents such as bile acids, which trigger a pathological rise in cytosolic Ca²^+^ within acinar cells. This sustained Ca²^+^ overload drives premature trypsinogen activation, mitochondrial dysfunction with ATP depletion, mitophagy, and NF-κB-mediated inflammatory signaling [3, 4]. The consequent autodigestion of acinar cells by activated proteases, compounded by energy failure and the onset of inflammatory cascades, precipitates acinar necrosis. This acinar injury, in turn, amplifies local inflammation and establishes a self-perpetuating vicious cycle that drives disease progression [5]. Of translational relevance, clinical studies have shown that pharmacological blockade of specific Ca²^+^ channels mitigates pancreatitis, highlighting the potential of targeting this early pathogenic checkpoint [6]. Consequently, early intervention targeting inflammatory mediator suppression and blockade of inflammatory amplification pathways constitutes a crucial therapeutic strategy for AP management.

Tumor necrosis factor α (TNFα) is a pivotal proinflammatory cytokine primarily secreted by activated immune cells, including macrophages and T cells. It plays a central role in inflammation, immune responses, and apoptosis [7, 8]. TNFα exerts its pleiotropic biological effects by engaging two distinct receptors: tumor necrosis factor receptor 1 (TNFR1) and TNFR2 [9]. While TNFR1 is ubiquitously expressed on most nucleated cells, TNFR2 exhibits restricted distribution, being predominantly limited to specific cell types such as immune cells and endothelial cells [7]. The TNF/TNFR1 axis is widely distributed and harbors a conserved death domain (DD) motif, primarily driving proinflammatory and tissue-degenerative responses. In contrast, TNFR2 mediates localized homeostatic effects, including cell survival and tissue regeneration [10, 11]. The TNF/TNFR1 axis is mediated through context-dependent homotypic DD interactions between TNFR1 trimers and downstream signaling components. This signaling cascade can induce the formation of at least three distinct complexes. Complex I formation at the plasma membrane activates NF-κB and MAPK/ERK pathway, modulating inflammatory responses and cell death to orchestrate immune regulation [12–14]. In contrast, Complex IIa and IIb drive apoptosis and necroptosis, respectively, clearing damaged or infected cells to sustain tissue homeostasis [7, 15]. The dynamic interplay between Complex I and II signaling ultimately dictates cellular fate decisions [16].

The TNF/TNFR1 axis is well-characterized in autoimmune and inflammatory disorders, including rheumatoid arthritis (RA) and inflammatory bowel disease (IBD). We previously showed that *Helicobacter pylori* infection activates the macrophage TNF/TNFR1 axis, inducing the NLRP3 inflammasome and thereby promoting gastritis [17]. Therapeutic targeting of this axis has emerged as a promising strategy to alleviate inflammation, and established drugs such as infliximab and adalimumab are effective in treating autoimmune diseases [18]. Clinical studies have consistently reported elevated serum TNF‑α levels in patients with acute pancreatitis (AP), with concentrations positively correlating with disease severity [19, 20]. However, the mechanisms by which the TNF/TNFR1 axis drives AP progression, and which TNF/TNFR1‑targeting inhibitors may exhibit therapeutic potential, remain unclear.

Here, we used wild‑type and *Tnfr1*^*–*^^*/*^^*–*^ mice in AP models to define the role of the TNF/TNFR1 axis in disease progression. Single‑cell and bulk RNA‑seq enabled precise mapping of the axis‑driven regulation of the pancreatic immune microenvironment, particularly in myeloid and neutrophil populations. Notably, we assessed three TNF/TNFR1 inhibitors for their preventive and therapeutic efficacy in experimental AP models, offering new insights for clinical treatment.

## Materials and methods

### Human serum sample collection and ethical statement

Human serum samples used in this study were obtained from The First Affiliated Hospital of Nanchang University. The collection and use of human samples were approved by the Clinical Research Ethics Committee of The First Affiliated Hospital of Nanchang University (Approval No. (2024) CDYFYYLK (06-039)). Written informed consent was obtained from all participants or their legal guardians prior to sample collection. Serum samples were obtained from two distinct cohorts: healthy donors (*n* = 8) and AP patients (*n* = 69). Patients were eligible for inclusion if they were aged 18–70 years, presented within 72 h of symptom onset, and were diagnosed with SAP based on the revised Atlanta criteria (2012) [21]. All samples were aliquoted and stored at −80 °C until subsequent analyses. Relevant information of AP patients can be found in Supplementary Table S11 and File S2.

### Animal study and approval

All animal procedures were conducted in accordance with institutional ethical guidelines and approved by the Animal Ethics Committee of the First Affiliated Hospital of Nanchang University (Approval No. CDYFY-IACUC-202306QR022). Male wild type (WT) and global *Tnfr1*^–/–^ mice on a C57BL/6 J background (GemPharmatech Co., Ltd.) at age 8–10 weeks (20–23 g) were used. Mice were housed under specific pathogen-free (SPF) conditions at 22–24 °C with a 12 h light/dark cycle and had ad libitum access to standard chow and water.

### Induction of AP mouse model and treatments

For the caerulein AP model, the mice received intraperitoneal (IP) injections of caerulein (100 μg/kg; Sigma-Aldrich, St. Louis, MO, USA) every hour for 10 h. Control mice received saline only. Mice were euthanized 24 h following the initial caerulein injection. The pancreas tissues and peripheral blood were collected for enzyme assays, cytokine measurement, and histopathological analysis.

For L-arginine AP, mice were given L-arginine IP at a total dose of 3 g/kg body weight (Sigma-Aldrich, Cat. A5006), divided into 3 injections at hourly intervals, while control mice were given saline. Samples were harvested 72 h after the first injection.

In the pancreatic duct ligation (LIG) pancreatitis model, mice were anesthetized and the distal part of the main pancreatic duct was ligated. After 48 h, the mice received a single IP injection of caerulein (50 μg/kg). Animals were euthanized 24 h later. Sham-operated mice underwent the same laparotomy procedure without duct ligation.

For therapeutic intervention studies, pomalidomide (Selleck, Houston, TX, USA, Cat. S1567), necrostatin-1 (Selleck, Cat. S8037), or Infliximab (Selleck, Cat. A2019) was administered by IP injection at low, medium, or high doses at 3 h and 12 h after AP induction. Stock and working solutions were prepared according to the manufacturer’s protocols.

### Cell culture and transfection

Murine RAW264.7 macrophages (ATCC, Manassas, VA, USA; TIB-71) were cultured in DMEM supplemented with 10% fetal bovine serum and 1% penicillin-streptomycin. Human HL-60 promyeloblasts (ATCC; CCL-240) were maintained in RPMI 1640 medium with the same supplements. Both cell lines were incubated at 37 °C in a humidified atmosphere containing 5% CO₂. To induce neutrophil-like differentiation, HL-60 cells were treated with 1.25% dimethyl sulfoxide (DMSO) for 5 days, with the medium refreshed every 2 days. Differentiation was confirmed on day 5 by CD11b expression via flow cytometry and by characteristic morphological changes.

For transient overexpression of Igkc and Marcksl1, cells were transfected with *Igkc* mRNA, *Marcksl1* mRNA, or a negative control mRNA using CALNP™ mRNA in Vitro Transfection Reagent (D-Nano Therapeutics, Beijing, China) according to the manufacturer’s instructions. Briefly, mRNA and transfection reagent were diluted separately in cell culture medium, mixed gently, and incubated for 10 min at room temperature to form mRNA-lipid complexes. These complexes were then added to the culture at a final mRNA concentration of 1 μg/mL.

### Pancreatic histology and immunohistochemistry

Pancreatic tissues were fixed in 4% formalin and embedded in paraffin. The specimens were then sectioned at 4 μm thickness and stained with hematoxylin and eosin (H&E) following standard protocols. Histological images were captured using a Nikon upright fluorescence microscope. Slides from all experimental groups were independently evaluated by two blinded observers. Each specimen was scored based on the severity and extent of edema, inflammatory cell infiltration, and acinar necrosis. The histological scoring system for pancreatic tissues was adopted from our previous study [22].

Immunohistochemistry was performed following our previously established protocol [23, 24]. Briefly, paraffin-embedded sections were deparaffinized, rehydrated, and subjected to antigen retrieval by boiling in 0.01 mol/L citrate buffer (pH 6.0) for 2 min. Endogenous peroxidase activity was blocked with 3% H₂O₂ for 8 min at room temperature, after which the sections were incubated overnight at 4 °C with primary antibodies against F4/80 (1:1000), MPO (1:1000), TNF-α (1:100), TNFR1 (1:50), and MARCKSL1 (1:500) (antibody details in Supplementary Table S1). After washing, the sections were incubated with the appropriate secondary antibody (ZSGB-BIO, China) for 30 min at 37 °C, and immunoreactivity was visualized using DAB. Nuclei were counterstained with hematoxylin, and the sections were dehydrated through graded ethanol, cleared in xylene, and mounted. Representative images were captured using a Nikon Eclipse microscope.

### Immunofluorescence

Immunofluorescence was conducted as previously described [25]. In brief, paraffin‑embedded pancreatic sections were deparaffinized in xylene, rehydrated in graded ethanol, and boiled in 0.01 M citrate buffer (pH 6.0) for 2 min for antigen retrieval. After cooling, sections were permeabilized with 0.3% Triton X‑100 (15 min, 37 °C) and blocked with 3% BSA in PBS (60 min, room temperature). Primary antibodies against AMYLASE, LYZ, S100A9, CITH3, MPO, TNFα, and F4/80 were applied overnight at 4 °C (details in Supplementary Table S1). After PBS washes, sections were incubated with Alexa Fluor‑conjugated secondary antibodies (1:500, Invitrogen) for 1 h at room temperature in the dark, followed by DAPI (Invitrogen) nuclear counterstaining. Sections were mounted with anti‑fade medium, and images were acquired using a confocal microscope.

### RNA extraction and quantitative real-time (PCR)

Total RNA was extracted from pancreatic tissues using TRIzol reagent (TIANGEN Biotech, Beijing, China) according to the manufacturer’s instructions. RNA purity and concentration were assessed by measuring the absorbance ratio at 260/280 nm using a Nanodrop spectrophotometer. After reverse transcription into cDNA, quantitative real-time PCR (qRT‑PCR) was performed on a QuantStudio 5 Real-Time PCR System (Life Technologies) using SYBR® Premix Ex Taq (RR820B; TaKaRa, Japan). Primer sequences were listed in Supplementary Table S2.

### Western blot

Protein extraction from pancreatic tissues was performed using RIPA lysis buffer supplemented with protease and phosphatase inhibitors. Protein concentrations were determined using a BCA assay kit (Thermo Fisher Scientific, USA). Equal amounts of protein were resolved by 10–12% SDS-PAGE, transferred onto PVDF membranes (Millipore, USA), and blocked with 5% non-fat milk for 1 h at room temperature. Membranes were then incubated with primary antibodies overnight at 4 °C, washed three times with TBST, and incubated with HRP-conjugated secondary antibodies for 1 h at room temperature. Protein signals were detected using an ECL substrate kit (Thermo Fisher Scientific, USA) and visualized with a Bio-Rad ChemiDoc chemiluminescence imaging system. Detailed information on all primary antibodies was provided in Supplementary Table S1. Uncropped western blot membranes are provided in File S1.

### ELISA assay

Mouse serum levels of TNF-α, IL-1β, and IL-6 were measured using commercial ELISA kits according to the manufacturer’s instructions. The kits were purchased from Elabscience Biotechnology Co., Ltd. (Wuhan, China) with the following product codes: TNF-α (Cat. E-EL-M3063), IL-1β (Cat. E-EL-M0037), and IL-6 (Cat. E-EL-M0044).

### Bulk-RNA sequencing analysis

Bulk RNA sequencing was performed to evaluate differences in transcriptional expression levels and biological process activation among groups. Briefly, pancreatic tissues from mice with acute pancreatitis were collected and homogenized. Total RNA was extracted, and library preparation was performed according to the instructions of the NEBNext Ultra II RNA Library Prep Kit. After sequencing, differential expression was analyzed using DESeq2 (v3.11); genes with a false discovery rate < 0.05 and |log₂ fold change | ≥1 were classed as significantly differentially expressed genes (DEGs). Functional enrichment analysis was performed using the clusterProfiler package. All differential genes were listed in Supplementary Tables S3, S9 and S10.

### Single-cell RNA sequencing(scRNA-seq) analysis

Raw sequencing reads were stored in FASTQ format. Quality control of raw data was performed using fastp (v0.23.4) to generate clean reads. The filtering procedure included: trimming 28 bp from read1; retaining read2 with average quality score >10; and removing read2 if five or more bases exceeded a quality threshold. Processed reads were then aligned to the mm9 reference genome using CellRanger (v7.1.0), which performed barcode error correction (against a whitelist), alignment with STAR (v2.7.8a), unique molecular identifier (UMI) deduplication, and gene expression matrix generation.

Cells were further filtered using Seurat (v4.3.0) with the following criteria: 200 ≤ nFeature_RNA ≤ 6000, and mitochondrial gene percentage <20%. The expression data were normalized and scaled using SCTransform (regularized negative binomial regression). Highly variable genes were selected for principal component analysis (PCA). The top principal components were used for uniform manifold approximation and projection (UMAP). Clustering was performed using a K‑nearest neighbor graph, refined by shared nearest neighbor (SNN), and partitioned with the Louvain algorithm. Cell clusters were annotated using SingleR (v2.0.0) with a reference dataset.

Differentially expressed genes (DEGs) between clusters were identified using the Wilcoxon rank‑sum test as implemented in the FindAllMarkers function of Seurat, with the following criteria: expression in ≥25% of cells in either cluster; Bonferroni‑adjusted *P* < 0.05; |log₂ (fold change) | >0.5. Functional enrichment analysis was performed using clusterProfiler (v4.16.0) to identify significantly enriched Gene Ontology (GO) terms, KEGG pathways, and Reactome pathways (adjusted *P* < 0.05). All details were listed in Supplementary Table S4–S8.

### Public sequencing data acquisition and processing

Publicly available single‑cell RNA‑sequencing data (GSE188819; 7 samples) were downloaded from the Gene Expression Omnibus (GEO). The raw count matrix was processed using Seurat (v4.3.0) in R. Cells with <200 genes or >20% mitochondrial read fractions were excluded. Data were normalized using LogNormalize, and the top 2000 highly variable genes were selected for subsequent analyses.

### Statistics

Quantitative data are reported as mean ± standard deviation (SD). Statistical evaluations were carried out using GraphPad Prism (V7.0). For comparison of two experimental groups, either parametric Student’s *t* test or nonparametric Mann–Whitney U test was applied as appropriate based on data distribution characteristics. Multigroup analyses employed one-way ANOVA with Fisher’s least significant difference post hoc testing. Bivariate correlations between microbial and metabolic variables were examined using Spearman’s rank-order correlation coefficient. Histopathological assessments were conducted independently by two board-certified pathologists under blinded conditions. The threshold for statistical significance was established at *P* < 0.05 (two-tailed).

## Results

### The TNF/TNFR1 axis is hyperactivated in murine models and patients with AP

**Fig. 1:**
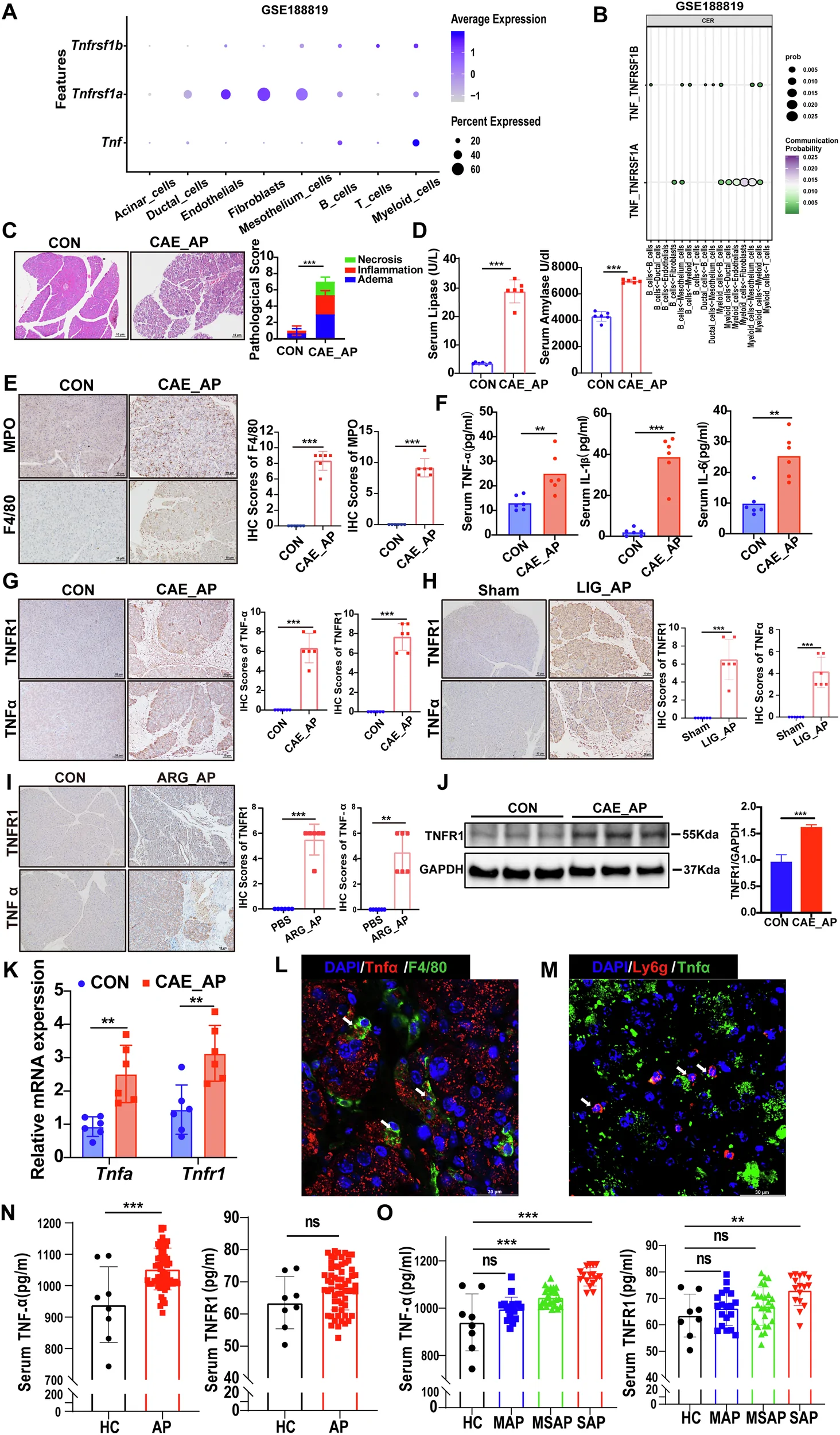
Activation of the TNF/TNFR1 axis in three AP animal models and AP patients. **A** Single cell RNA sequencing illustrating the expression of tumor necrosis factor-α *(Tnfα)*, tumor necrosis factor receptor superfamily member 1a *(Tnfrsf1a)*, and member 1b *(Tnfrsf1b)* genes across various cell types in caerulein induced AP. **B** Cellular communication analysis reveals enhanced ligand-receptor interaction probability between *Tnfα* and *Tnfrsf1b*. **C** Representative H&E-stained pancreatic tissue sections (scale bar: 10 μm) and pathological scoring in control and caerulein-induced AP (CAE_AP) model mice. **D** Serum lipase and amylase levels in control and CAE_AP mice. **E** Immunohistochemical (IHC) assessment of neutrophil infiltration (myeloperoxidase, MPO) and macrophage accumulation (F4/80) in pancreatic tissues (scale bar = 10 μm) with quantitative scoring. **F** The levels of serum inflammatory cytokines (TNFα, IL-1β, and IL-6) in control and CAE_AP mice. **G** IHC staining and scoring of TNFα and TNFR1 in pancreatic tissues in control and CAE_AP mice. **H** IHC staining and scoring of TNFα and TNFR1 expression in pancreatic tissues of control and pancreatic duct ligation-induced AP (LIG_AP) mice. **I** IHC staining and scoring of TNFα and TNFR1 in pancreatic tissues from sham-operated and L-arginine-induced AP (ARG_AP) groups. **J** Western blot analysis and quantification of TNFR1 protein expression in pancreatic tissues from control and CAE_AP model mice. **K** Quantitative reverse transcription polymerase chain reaction (qRT-PCR) measurement of *Tnfα* and *Tnfr1* transcript levels. **L** Immunofluorescence co-localization of TNFα (red) and F4/80 (green). **M** Immunofluorescence co-localization of TNFα (green) and LY6G (red). **N** Enzyme-linked immunosorbent assay (ELISA) quantification of serum TNFα and TNFR1 levels in serum from AP patients. **O** Comparative analysis of TNFα and TNFR1 protein levels among healthy controls and AP patients stratified by disease severity. *P < 0.05, **P < 0.01, and ***P < 0.001 were considered significant. Data were expressed as the means ± SD. Data represent mean ± standard deviation (SD) of at least three independent experiments.

TNFα, a key inflammatory mediator in acute pancreatitis (AP), signals through two distinct receptors: TNFR1 (encoded by *Tnfrsf1a*) and TNFR2 (encoded by *Tnfrsf1b*). Analysis of ScRNA-seq dataset GSE188819 from an experimental AP model induced by caerulein revealed that *Tnf* was predominantly expressed in immune cells. In contrast, its receptor *Tnfr1* was widely expressed across multiple pancreatic cell types, whereas *Tnfr2* was expressed at low levels throughout pancreatic tissues (Fig. 1A). Cell-cell communication analysis further indicated that the TNF signaling network, primarily mediated by the TNF/TNFR1 axis, was highly activated in AP (Fig. 1B). To investigate the expression of TNF and TNFR1 in AP, we established three experimental AP models: caerulein-induced AP (CAE_AP), pancreatic duct ligation-induced AP (LIG_AP), and L-arginine-induced AP (ARG_AP) model. All models were successfully constructed based on histopathological features such as pancreatic edema, inflammatory cell infiltration, and partial acinar cell necrosis, as shown by H&E staining (Fig. 1C, S1A, B). Consistent with human AP manifestations, the CAE_AP model exhibited significant increases in serum lipase and amylase levels (Fig. 1D). Substantial inflammatory infiltration was further confirmed by IHC staining, with elevated expression of the macrophage marker F4/80 and neutrophil marker MPO (Fig. 1D, S1C, D). Similarly, mice with AP showed markedly higher serum levels of pro-inflammatory cytokines TNFα, IL-1β, and IL-6 compared to controls (Fig. 1F). Notably, we observed increased expression of TNFα and TNFR1 in all three experimental AP models relative to control mice (Fig. 1G–I). These observations were corroborated by Western blot analysis (Fig. 1J). Furthermore, qRT-PCR analysis indicated upregulation of *Tnfα* and *Tnfr1* mRNA in AP mice (Fig. 1K). Analysis of clinical serum samples showed significantly elevated TNFα levels in AP patients compared with healthy controls, with levels increasing in accordance with disease severity (Fig. 1N). Multivariate analysis further confirmed that both TNFα and TNFR1 levels were independently associated with the severity of AP (Supplemental File S2). Consistently, TNFR1 levels were higher in serum from severe AP (SAP) patients than in healthy controls (Fig. 1O). Taken together, these results confirm activation of the TNF/TNFR1 signaling axis in AP progression.

### TNFR1 knockout attenuates pancreatic inflammation in experimental AP

**Fig. 2:**
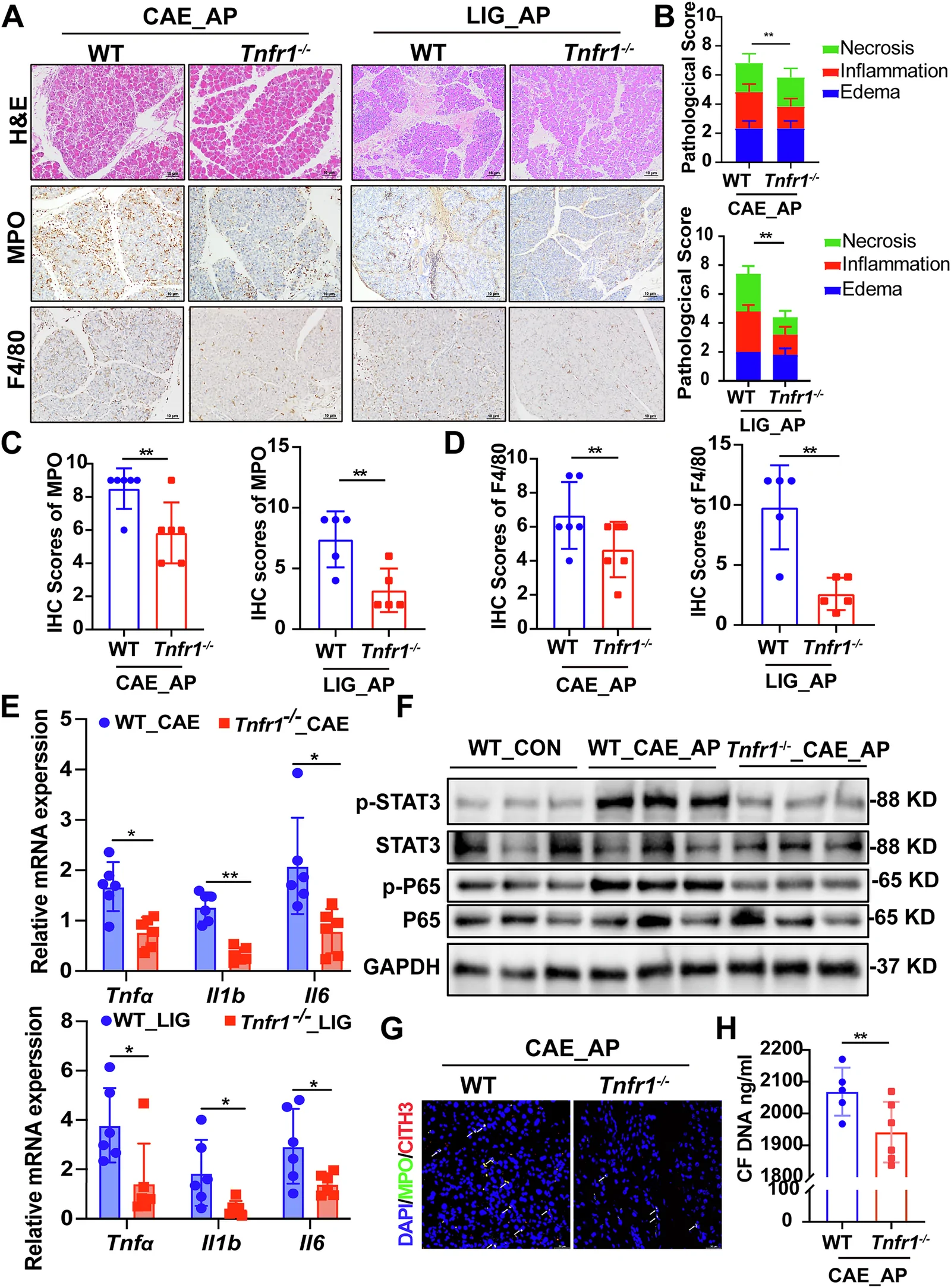
*Tnfr1* deletion attenuates inflammatory responses in two acute pancreatitis animal models: caerulein-induced (CAE_AP) and duct ligation (LIG_AP). **A** Representative H&E and IHC analysis of pancreatic tissues from wild-type (WT) and *Tnfr1*^–/–^ mice following caerulein- or duct ligation-induced AP. H&E staining reveals tissue damage, while IHC detects neutrophil infiltration (myeloperoxidase, MPO) and macrophage accumulation (F4/80) (scale bar = 10 μm). **B** Quantitative scoring of pancreatic injury severity (H&E-stained sections from **A**). Quantitative assessment of MPO (neutrophils, **C**) and F4/80 (macrophages, **D**) by IHC scoring in caerulein - and duct ligation-induced AP models. **E** qRT-PCR analysis of inflammatory cytokine mRNA levels (*Il1b*, *Il6*, *Tnf*) in pancreatic tissues from WT and *Tnfr1*^–/–^ mice. **F** Western blot detection of P65, STAT3, and their phosphorylated forms (p-P65, p-STAT3) in pancreatic tissues from control or caerulein-induced AP mice (WT vs. *Tnfr1*^–/–^). **G** Immunofluorescence detection of neutrophil extracellular traps (NETs) via MPO (green) and citrullinated histone H3 (CitH3, red) co-localization. **H** Quantification of serum cell-free DNA (cfDNA, NETs marker) by fluorometric assay. Statistical significance was assessed by one-way ANOVA (for multi-group comparisons) or two-tailed Student’s *t* test (for two-group comparisons), with *P < 0.05, **P < 0.01, and ***P < 0.001 considered significant. Data are presented as mean ± SD from three independent experiments.

To investigate the role of the TNF/TNFR1 axis in the pathogenesis of AP, we generated conventional global *Tnfr1* knockout (*Tnfr1*^–/–^) mice. Successful ablation of *Tnfr1* was confirmed by genotyping and qRT-PCR analysis (Fig. S2A, B). Then, AP was induced in both WT and *Tnfr1*^–/–^ mice using two established models: caerulein injections and pancreatic duct ligation. Histopathological analysis indicated a significant reduction in pancreatic inflammation in *Tnfr1* deficient mice than in WT controls following the AP induction via caerulein injection (CAE_AP) or pancreatic duct ligation (LIG_AP) (Fig. 2A, B). Consistently, the neutrophil marker MPO and macrophage marker F4/80 was markedly reduced in the pancreatic tissues of *Tnfr1*^–/–^ mice with AP compared to WT controls (Fig. 2A, C, D). However, serum amylase and lipase levels remained comparable between genotypes (Fig. S2C). Further supporting this notion, qRT-PCR analysis of pancreatic tissue revealed a significant downregulation of pro-inflammatory cytokines, including *Tnfα*, *Il1b*, and *Il6*, in both CAE- and LIG-induced AP models of *Tnfr1*^−/−^ mice compared to WT group (Fig. 2E). However, serum TNFα levels showed no statistically significant difference between the two groups (Fig. S2D). We next assessed the NF-κB and STAT3 pathways, which are activated by TNF/TNFR1 signaling and play a well-established role in proinflammatory response. Pancreatic P65 and STAT3 phosphorylation was markedly elevated in CAE_AP WT mice but was abolished in *Tnfr1*-deficient mice with AP (Fig. 2F, E). Furthermore, immunofluorescence staining for the markers CITH3 and MPO was performed to assess the neutrophil extracellular traps (NETs) formation. Notably, there was a pronounced reduction in CITH3^+^MPO^+^ NETs structures in the pancreatic tissues of *Tnfr1*^–/–^ mice with AP relative to their WT counterparts (Fig. 2G). In agreement with these data, serum cfDNA levels were also significantly decreased in *Tnfr1*^–/–^ AP mice (Fig. 2H). Collectively, these data revealed that *Tnfr1* deficiency significantly attenuates inflammatory infiltration during AP progression.

### Single-cell analysis uncovers altered immune landscape in TNFR1-depleted AP mice

**Fig. 3:**
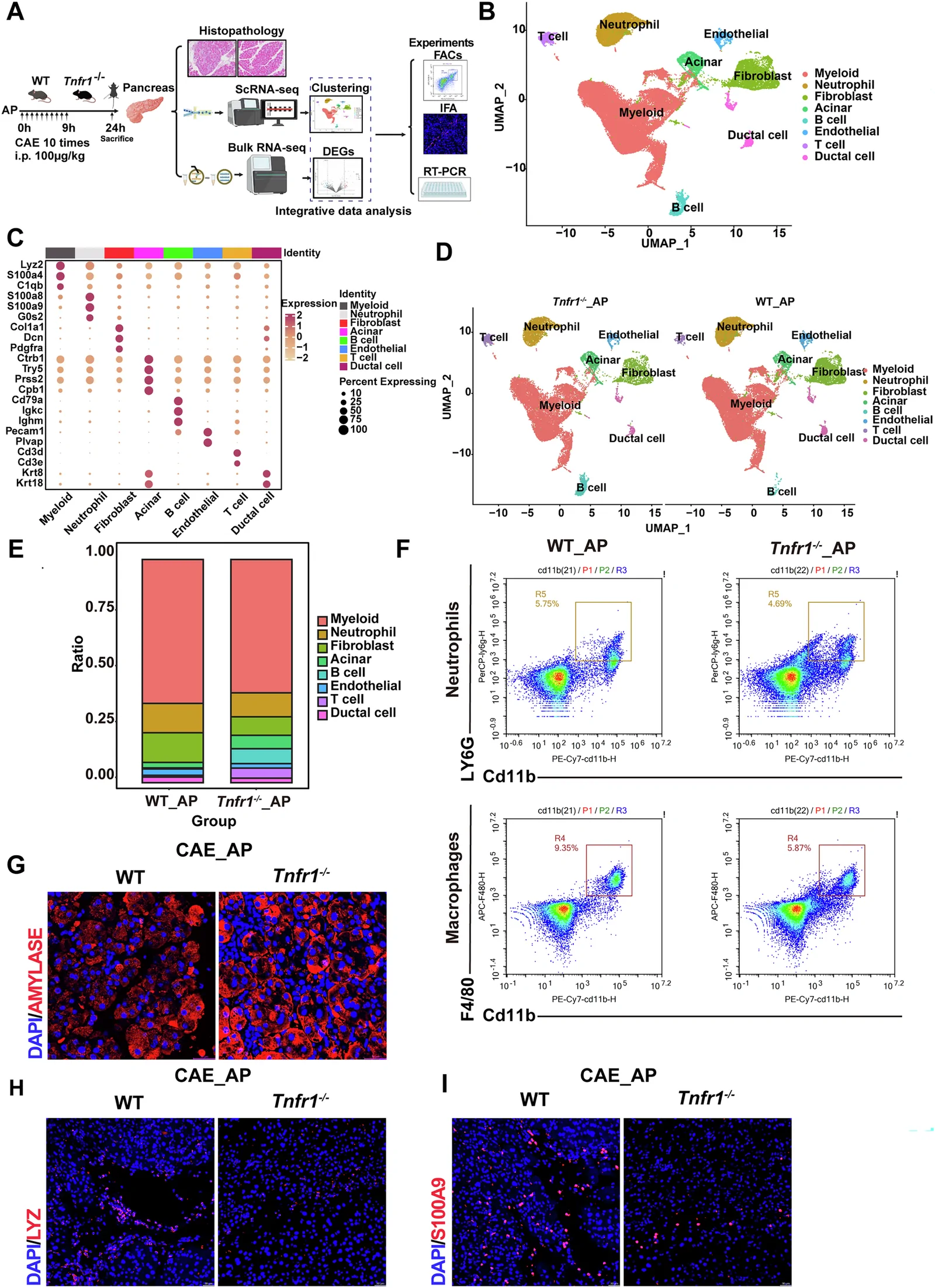
*Tnfr1* deficiency alters inflammatory cell profiles in acute pancreatitis revealed by single-cell RNA sequencing (scRNA-seq). **A** Schematic workflow of scRNA-seq experimental design and computational analysis pipeline. **B** Uniform Manifold Approximation and Projection (UMAP) visualization of all sequenced cells, colored by annotated cell type clusters (*n* = 52,277). **C** Dot plot of lineage-defining marker genes across the 8 identified cell populations: dot size represents expression prevalence; color indicates mean normalized expression. **D** UMAP plots stratified by genotype, highlighting differential cell distribution between WT and *Tnfr1*^–/–^ mice. **E** Stacked bars showing proportional abundance of each cell type in WT versus *Tnfr1*^–/–^ mice. **F** Flow cytometry quantification of pancreatic neutrophil (CD11b^+^ Ly6G^+^) and macrophage (CD11b^+^ F4/80^+^) frequencies. **G**–**I** Representative immunofluorescence images of pancreatic sections stained for acinar cells (Amylase^+^, green), macrophages (Lyz^+^, red), and neutrophils (S100A9^+^, white). Nuclei counterstained with DAPI (blue; scale bar = 50 μm).

To define the comprehensive cellular mechanisms by which *Tnfr1* deficiency modulates inflammatory responses, we conducted scRNA-seq to compare the cellular heterogeneity and phenotype-specific alterations across various cell populations between WT and *Tnfr1*^–/–^ mice with CAE-induced AP (Fig. 3A). Unsupervised clustering of transcriptomes identified eight major cell populations (Fig. 3B), annotated based on specific cell markers: Myeloid cells (*Lyz2*, *S100a4*, *C1qb*), Neutrophils (*S100a8*, *S100a9*, *G0s2*), Fibroblasts (*Col1a1*, *Dcn*, *Pdgfra*), Acinar cells (*Ctrb1*, *Try5*, *Prss2*, *Cpb1*), B cells (*Cd79a*, *Igkc*, *Ighm*), Endothelial cells (*Pecam1*, *Plvap*), T cells (*Cd3d*, *Cd3e*) and Ductal cells (*Krt8*, *Krt18*) (Fig. 3C). The top five marker genes per cell type were shown in the heatmap across all populations (Fig. S3A).

Comparative analysis revealed a marked reduction in myeloid and neutrophil populations in *Tnfr1*^*–/–*^ mice, along with an expansion of the acinar cell compartment (Fig. 3D, E, S3B), consistent with our previous observations of attenuated inflammation. Flow cytometry confirmed decreased infiltration of neutrophils (CD11b^+^Ly6G^+^) and macrophages (CD11b^+^F4/80^+^) in *Tnfr1*^*–/–*^ mice with AP compared to the WT AP group (Fig. 3F). In agreement with scRNA-seq findings, immunofluorescence staining showed a significant increase in the acinar marker Amylase, alongside reduced expression of the myeloid marker LYZ and the neutrophil marker S100A9, in pancreatic tissues of *Tnfr1*-deficient AP mice relative to WT controls (Fig. 3G–I). These results align with earlier data demonstrating that *Tnfr1* knockout leads to a substantial decrease in immune cells infiltration in AP models.

### Multi-omics integration reveals that *Tnfr1* ablation impairs neutrophil and macrophage recruitment and inflammatory cytokine release

**Fig. 4:**
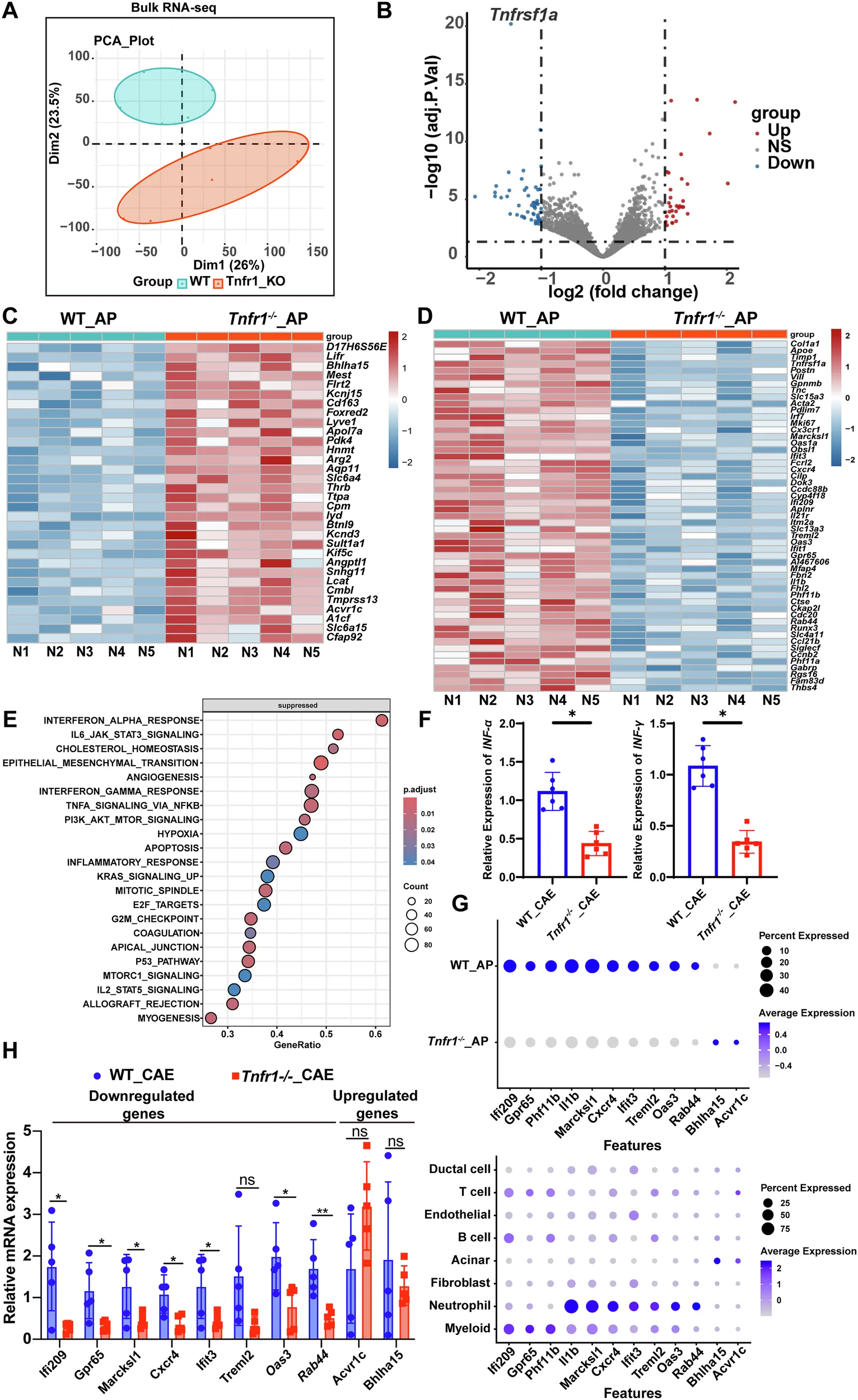
Multi-omics analysis identifies *Tnfr1*-dependent neutrophil/macrophage dysregulation and cytokine suppression. **A** Principal component analysis (PCA) of bulk RNA-seq data in WT and *Tnfr1*^–/–^ transcriptomes, illustrating genotype-driven clustering (WT vs. *Tnfr1*^–/–^). **B** Volcano plot of differentially expressed genes (DEGs: log_2(_fold change) ≥1, *P*.*adjust* < 0.05) between WT and *Tnfr1*^–/–^ mice, red highlights statistically significant DEGs. Heatmaps of genotype-specific DEGs: *Tnfr1*^–/–^ upregulated genes (**C**) and *Tnfr1*^−/−^ downregulated genes (**D**) (Z-score normalized expression). **E** GSEA based on significantly differentially expressed genes (WT vs. *Tnfr1*^–/–^). **F** Transcriptional expression levels of IFN-α, and IFN-γ. **G** Dot plot of consensus DEGs from scRNA‑seq and bulk RNA‑seq, displayed across groups and cell types (rows: prioritized genes; columns: groups/cell types). Dot size denotes expression prevalence; color represents mean normalized expression. **H** qRT-PCR validation of 11 prioritized DEGs in pancreatic tissues from WT and *Tnfr1*^–/–^ mice. *P < 0.05, **P < 0.01, and ***P < 0.001 were considered significant. Data are presented as mean ± SD from three independent experiments.

To comprehensively characterize the transcriptional landscape, bulk RNA sequencing was performed on pancreatic tissues from WT and *Tnfr1*^–/–^ mice. This integrated multi-omics approach enabled more precise identification of inflammation-associated key genes through cross-platform validation. Principal component analysis of the bulk transcriptome data revealed high intra-group reproducibility and substantial inter-group separation (Fig. 4A). Volcano plot analysis identified numerous significantly differentially expressed genes (DEGs), with heatmaps displaying genes upregulated or downregulated in *Tnfr1*^–/–^ mice (Fig. 4C, D, Supplementary Table S3). The gene set enrichment analysis (GSEA) results indicated that inflammatory pathways, including the IL6_JAK_STAT3 pathway, the TNFα/NF-κB pathway, and interferon (IFN) pathways, were downregulated in *Tnfr1*^–/–^ mice (Fig. 4E, Supplementary Table S4). Downregulation of IFN‑α and  IFN‑γ in *Tnfr1*^*–/–*^ mice was further confirmed by qRT‑PCR (Fig. 4F).

Cross-referencing these DEGs with scRNA-seq data, we identified 11 genes (*Ifi209*, *Gpr65*, *Phf11b*, *Marcksl1*, *Cxcr4*, *Ifit3*, *Treml2*, *Oas3*, *Rab44*, *Bhlha15*, and *Acvr1c*) showing consistent expression trends across both sequencing platforms (Fig. 4G, S4A). Among the *Tnfr1*-dependent genes identified, a substantial proportion were functionally linked to IFN pathways, suggesting that the TNF/TNFR1 axis exacerbates AP inflammation, at least in part, by potentiating IFN-α, IFN-γ production and downstream inflammatory responses. Upregulated genes in the WT group were functionally enriched in inflammatory and immune responses. However, *Bhlha15* and *Acvr1c* were upregulated in acinar cells from *Tnfr1*^–/–^ group, may contribute to acinar cell homeostasis. Cell‑type‑specific mapping showed that in wild‑type AP mice, upregulated DEGs were enriched in myeloid cells (*Ifi209*, *Gpr65*, *Phf11b*) and neutrophils (*Marcksl1*, *Cxcr*4, *Ifit3*, *Treml2*, *Oas3*, *Rab44*). By contrast, in *Tnfr1*‑deficient AP mice, the significantly upregulated genes *Bhlha15* and *Acvr1c* were mainly expressed in acinar cells (Fig. S4A). Most pro‑inflammatory DEGs were confirmed by qRT‑PCR, with the exception of *Phf11b* and *Treml2* (Fig. 4H). These findings reveal a molecular signature in myeloid cells and neutrophils regulated by the TNF/TNFR1 signaling axis during AP progression.

### *Tnfr1* deficiency reprograms neutrophils into an inflammation-suppressive phenotype

**Fig. 5:**
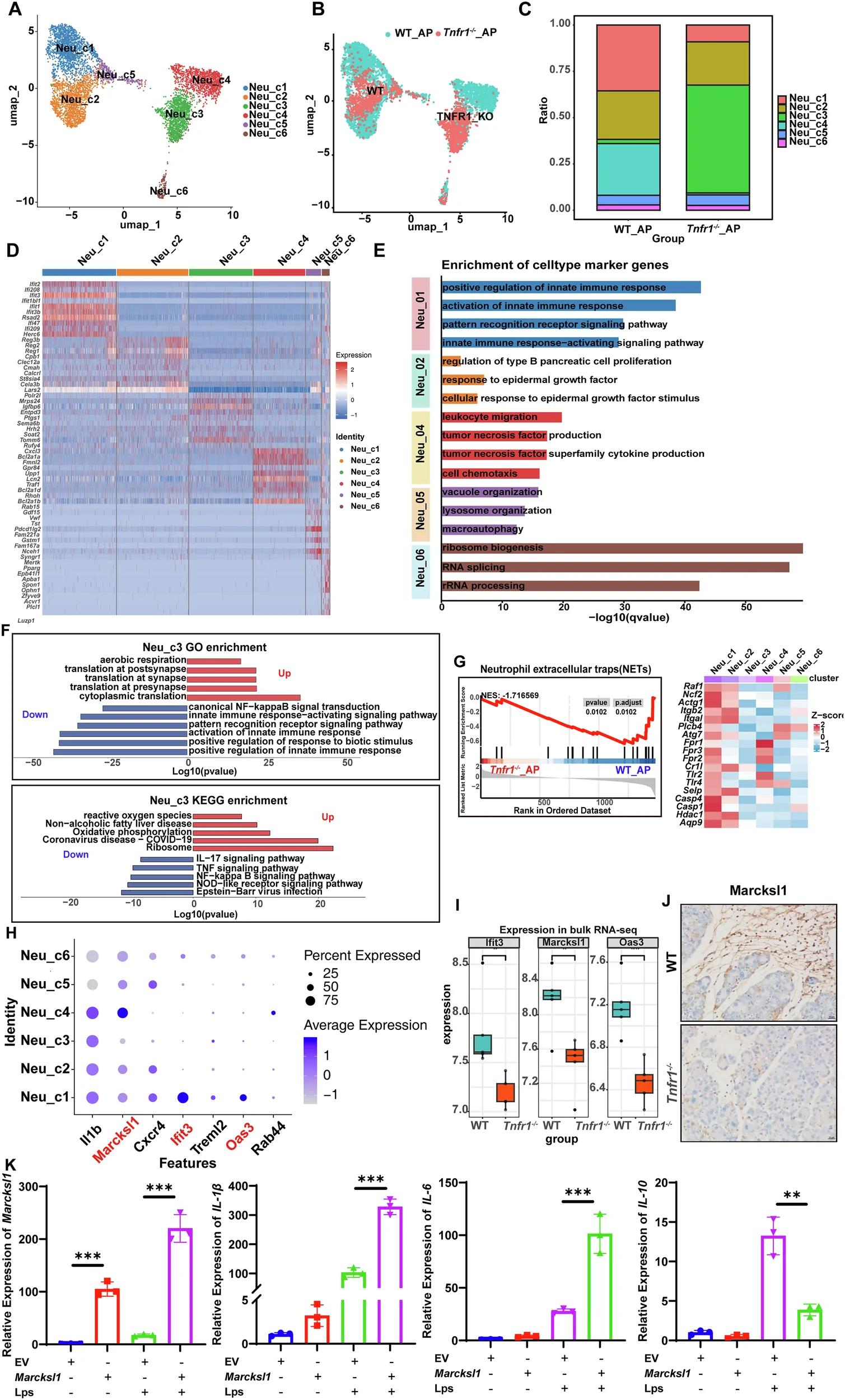
*Tnfr1*^–/–^ neutrophils exhibit reduced *Marcksl1, Ifit3, and Oas3* expression and acquire inflammatory suppression properties. **A** UMAP visualization of neutrophil subclusters from scRNA-seq data (colors denote subsets Neu_01-06). **B** Genotype-stratified UMAP plots of neutrophil subclusters between WT and *Tnfr1*^–/–^ mice. **C** Quantification of neutrophil (Neu_01-06) subset proportions across genotypes. **D** Heatmap of top 10 marker genes for neutrophil subclusters. **E** GO term enrichment analysis of Neu_01/02/04/05/06 functional pathways. **F** Integrated GO/KEGG pathway analysis revealing inflammatory suppression functions of Neu_03. **G** Neutrophil extracellular trap (NET) signatures. *Upper*: Heatmap of NETs-associated genes across subclusters; *Lower*: GSEA of NETs-related gene sets in WT vs. *Tnfr1*^*–/–*^ neutrophils. **H** Expression dynamics of *Marcksl1*, *Ifit3*, *Oas3*, *Cxcr4*, *Treml2*, and *Rab44* across subclusters (dot size: detection rate; color: mean log-normalized expression). **I** Validation of *Marcksl1*, *Ifit3*, and *Oas3* expression via bulk RNA-seq (isolated neutrophils; mean ± SD; *n* = 5). **J** Immunohistochemical quantification of MARCKSL1 protein. **K** Transcriptional levels of *Marcksl1* and inflammatory cytokines in HL-60 cells overexpressing *Marcksl1*. *P < 0.05, **P < 0.01, and ***P < 0.001 were considered significant. Data were expressed as the means ± SD.

To investigate the role of the TNF/TNFR1 signaling axis in regulating neutrophil heterogeneity and molecular profiles, we performed dimensionality reduction and clustering analyses. This identified six transcriptionally distinct neutrophil subclusters: Neu_C1 (high expression of *Lfit* family genes *Lfit2* and *Lfit3*), Neu_C2 (*Reg3b*^*+*^*Reg2*^*+*^), Neu_C3 (*Igfbp6*^*+*^*Ptgs1*^*+*^), Neu_C4 (*Cxcl3*^*+*^), Neu_C5 (*Rab15*^*+*^), and Neu_C6 (*Mertk*^*+*^*Pparg*^*+*^) (Fig. 5A, D). Comparative UMAP visualization revealed marked heterogeneity in neutrophil expression profiles between WT and *Tnfr1*^–/–^ mice with AP. Notably, the proportion of the Neu_C3 subcluster was significantly increased in pancreatic tissues of *Tnfr1*^−/−^ mice compared with WT controls, whereas Neu_C1 and Neu_C4 were the predominant subsets in WT mice (Fig. 5B, C). In WT AP mice, Neu_C1 and Neu_C4 were enriched in innate immune response signaling pathways and in pathways related to TNF production, chemokine axis, and leukocyte migration, respectively (Fig. 5E). In contrast, in *Tnfr1*^−/−^ AP mice, the abundantly present Neu_C3 subcluster exhibited significant downregulation of pro-inflammatory immune pathways, including pattern recognition receptor signaling, innate immune response, and NF-κB signaling, suggesting that expansion of this subcluster mediates the attenuation of AP upon *Tnfr1* deficiency (Fig. 5F). Similarly, the NETs pathway, a microbicidal structure of antimicrobial proteins and DNA released by activated neutrophils during acute inflammation, was also markedly downregulated in *Tnfr1*^–/–^ AP mice compared with WT AP controls (Fig. 5G, S5A).

We next integrated single‑cell and bulk RNA‑sequencing analyses to identify neutrophil‑enriched differentially expressed genes with concordant expression trends across datasets. The results revealed that *Lfit3* and *Oas3* were differentially expressed in the Neu_C1 subcluster, which predominates in WT AP mice, whereas Marcksl1 was differentially expressed in the Neu_C2 subcluster (Fig. 5H). In pancreatic tissues from *Tnfr1*‑deficient AP mice, the mRNA levels of all three genes were significantly lower than those in WT AP controls (Fig. 5I). Consistent with its known role in regulating cytoskeletal dynamics required for reactive oxygen species production and NETs, MARCKSL1 showed neutrophil‑specific localization by immunohistochemistry and was markedly reduced upon *Tnfr1* knockout (Fig. 5J).

To functionally assess the role of MARCKSL1 in neutrophil‑driven inflammation, we overexpressed MARCKSL1 in HL‑60 promyelocytic cells that had been differentiated into neutrophil‑like cells using DMSO. LPS stimulation strongly upregulated the pro‑inflammatory cytokines *IL1β* and *IL6* while downregulating the anti‑inflammatory cytokine *IL10*. MARCKSL1 overexpression further potentiated both of these effects (Fig. 5K). Together, these data suggest that the TNF/TNFR1 signaling axis promotes AP pathogenesis in neutrophils by enhancing MARCKSL1-mediated pro‑inflammatory signaling.

### TNFR1 deficiency increases *Igkc*^*+*^ myeloid cells with inflammation-suppressive function to alleviate AP

**Fig. 6:**
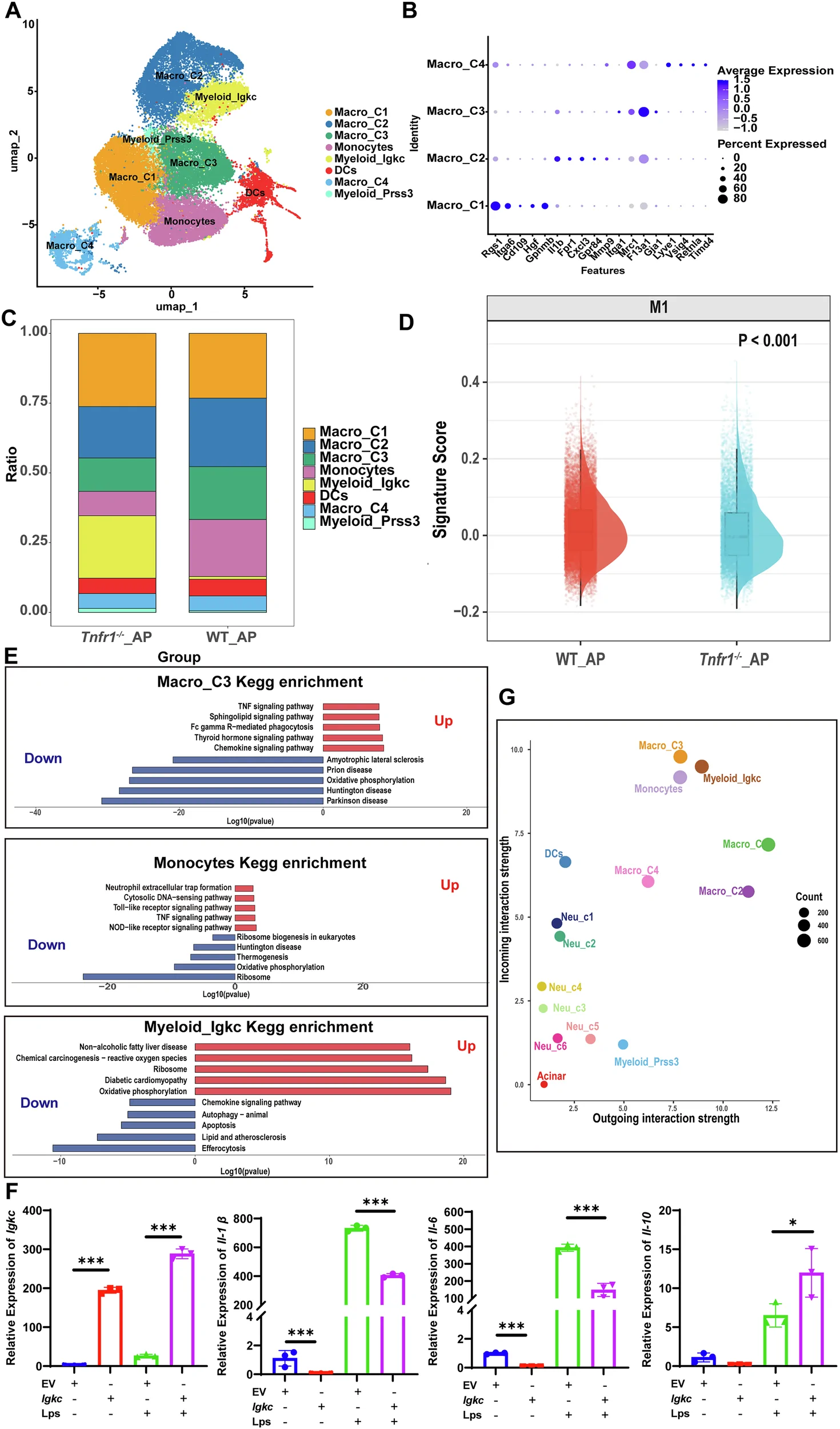
A *Tnfr1*^–/–^ dependent Myeloid_Igkc subpopulation attenuates inflammatory signaling in acute pancreatitis. **A** UMAP visualization of myeloid clusters from scRNA-seq data (colors: Macrophage/Monocyte subsets). **B** Dot plot of signature gene expression across 4 macrophage subsets (dot size: detection rate; color: Z-score). **C** Quantification of myeloid subset frequencies showing relative expansion of *Myeloid_Igkc* in *Tnfr1*^–/–^ mice. **D** M1 score of WT_AP and *Tnfr1*^–/–^_AP group. **E** KEGG pathway enrichment reveals distinct immunological functions: *Upper*: pro-inflammatory pathways in Macro_C3 and Monocytes; *Lower*: Inflammation-suppressive signatures in Myeloid_Igkc. **F** Based on cell communication analysis (CellChat), the bubble plot shows the communication activity among acinar cells, neutrophils, and myeloid cell subpopulations. **G** Transcriptional expression levels of *Igkc* and inflammatory cytokines in RAW264.7 cells overexpressing *Igkc*. *P < 0.05, **P < 0.01, and ***P < 0.001 were considered significant. Data were expressed as the means ± SD.

We previously found that myeloid cells are reduced in *Tnfr1*-deficient mice during acute pancreatitis. High‑resolution mapping of the myeloid compartment subsequently resolved eight subclusters, including four macrophage subsets: Macro_C1 (*Rgs1*^*+*^*Itga6*^*+*^), Macro_C2 (*Il1b*^*+*^*Cxcl3*^*+*^), Macro_C3 (*F13a1*^*+*^*Itga1*^*+*^), and Macro_C4 (*Lyve1*^*+*^*Vsig4*^*+*^) (Fig. 6B). The remaining subclusters comprised *Ly6c*^*+*^ monocytes, *Cd209a*^*+*^*Flt3*^*+*^ dendritic cells, and two novel myeloid subsets characterized by *Igkc* and P*rss3* expression, respectively (Fig. 6A, S6A, B). Comparative analysis revealed striking distribution differences: the *Myeloid_Igkc* subcluster was almost exclusively derived from *Tnfr1*^–/–^ mice, while Macro_C3 and Monocytes predominated in the WT group (Fig. 6C). Macrophage polarization scoring indicated markedly reduced M1 polarization in *Tnfr1*^−/−^ AP mice versus wild‑type AP controls, aligning with the attenuated inflammatory phenotype upon TNFR1 loss (Fig. 6D). The Macro_C3 subcluster was linked to enrichment of inflammation‑amplifying pathways including TNF and chemokine signaling. The monocyte subcluster is similarly enriched in NETs, TLR, NOD‑like, and TNF signaling pathways. Conversely, the expanded Myeloid_Igkc subcluster in *Tnfr1*^–/–^ AP mice downregulated efferocytosis, lipid metabolism, and chemokine signaling pathways (Fig. 6E).

We next asked whether Igkc confers inflammation-suppressive functions in myeloid cells. To address this, we overexpressed Igkc in RAW264.7 mouse macrophages and then challenged the cells with LPS. qRT‑PCR showed that *Igkc* overexpression markedly inhibited the LPS‑driven upregulation of the pro‑inflammatory cytokines *Il1b* and *Il6* and, conversely, increased the expression of the anti‑inflammatory cytokine *Il10* (Fig. 6F). To interrogate intercellular communication among acinar cells, myeloid cells, and neutrophils in the TNF/TNFR1‑shaped pancreatic microenvironment during acute pancreatitis, we performed CellChat analysis (Fig. S6C). This identified macrophages and Igkc^+^ myeloid cells as the major outgoing signaling hubs, with neutrophils and acinar cells as the primary targets (Fig. 6G). Collectively, these data indicate that TNFR1 deficiency reshapes the myeloid compartment, resulting in the expansion of inflammation-suppressive *Igkc*^*+*^ myeloid cells that counteract AP pathogenesis.

### Prophylactic blockade of TNF/TNFR1 axis by pomalidomide ameliorates inflammation and acinar cell damage in AP

**Fig. 7:**
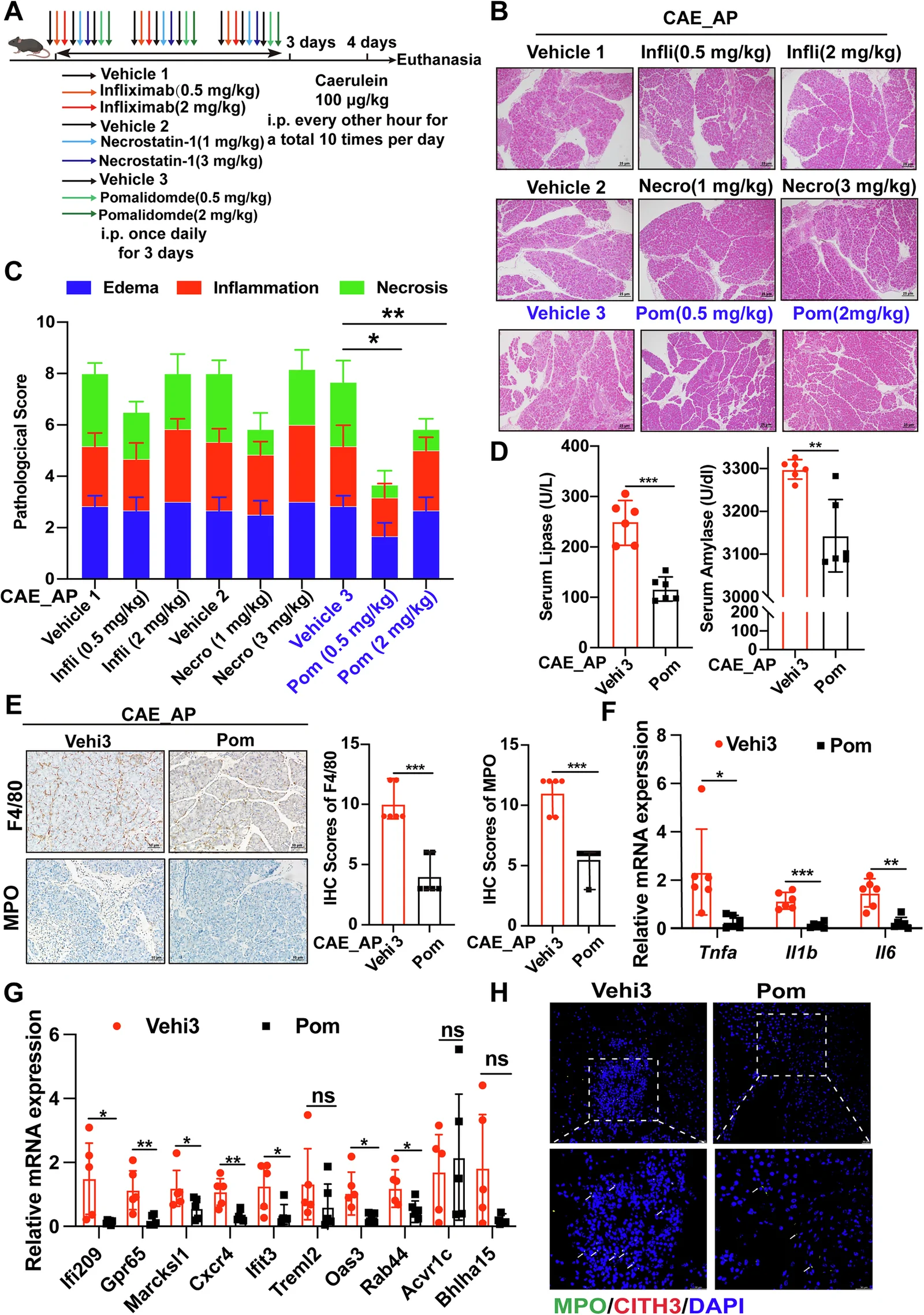
Three TNF/TNFR1 pharmacological blockades attenuate inflammation and acinar damage in acute pancreatitis. **A** Treatment timeline: Mice received TNFα inhibitors (pomalidomide 0.5 mg/kg, necrostatin-1 10 mg/kg, infliximab 5 mg/kg) or vehicle before caerulein-induced AP. Pomalidomide showed the most promising effect. **B** Representative H&E-stained pancreatic sections from three inhibitor-treated groups and vehicle controls. **C** Histopathological scoring of pancreatic injury (∑ edema/necrosis/inflammation; mean ± SD; *n* = 6). **D** Serum amylase and lipase levels following pomalidomide optimization (0.5 mg/kg dose selected for downstream assays). **E** IHC analysis and quantification of MPO (left) and macrophage F4/80 infiltration (right). **F** qRT-PCR measurement of pancreatic inflammatory cytokines. **G** Expression profiling of neutrophil-associated genes (*Ifi209*, *Gpr65*, *Phf11b*, *Marcksl1*, *Cxcr4, Ifit3*, *Treml2*, *Oas3*, *Rab44*, *Bhlha15*, and *Acvr1c*) by qRT-PCR. **H** IF co-staining of MPO (green) and CitH3 (red) to assess NET formation, scale bar = 50 μm. *P < 0.05, **P < 0.01, and ***P < 0.001 were considered significant. Data were expressed as the means ± SD.

We previously established the TNF/TNFR1 axis as a key driver of AP. To bridge these findings with clinical therapeutic potential, we investigated three distinct inhibitors of the TNF/TNFR1 axis: infliximab, necrostatin-1, and pomalidomide. Drugs were administered daily for 3 days prior to AP induction to establish TNF /TNFR1 axis inhibition before disease modeling (Fig. 7A). Among the three inhibitors, pomalidomide showed the most significant ameliorative effects on AP, as evidenced by reduced acinar cell necrosis and diminished inflammatory responses (Fig. 7B, C). Pomalidomide at 0.5 mg/kg was determined as the optimal dose and used for subsequent experiments. Compared with the vehicle control group, pomalidomide treatment significantly reduced serum amylase and lipase levels (Fig. 7D). Consistently, in pomalidomide‑treated experimental AP mice, immunohistochemical staining for MPO and F4/80 revealed decreased neutrophil and macrophage infiltration, respectively, accompanied by reduced mRNA levels of the inflammatory cytokines *Tnfα*, *Il1b*, and *Il6* (Fig. 7E, F). Although the inhibitors infliximab and necrostatin-1 could slightly reduce the recruitment of inflammatory cells and decrease the expression of MPO and F4/80 in immunohistochemical staining, their effects were not significant compared with pomalidomide (Fig. S7A–D). Concurrently, immunofluorescence co‑staining for MPO and CITH3 revealed that neutrophil extracellular trap release was significantly reduced in AP mice following pomalidomide treatment (Fig. 7H). We next sought to determine whether the changes in the pancreatic molecular expression profile induced by the TNF/TNFR1 inhibitor pomalidomide in acute pancreatitis mirror those observed in *Tnfr1*^–/–^ mice. Consistent with the findings in *Tnfr1*‑deficient mice, pomalidomide treatment significantly suppressed the expression of key pro‑inflammatory factors in the pancreatic tissue of AP mice, including *Marcksl1*, *Cxcr4*, *Lfit3*, and *Oas*3 (Fig. 7G). Collectively, these data demonstrate that the TNF/TNFR1 axis inhibitor pomalidomide exerts a robust protective effect against acute pancreatitis.

### Pomalidomide exerts a pronounced therapeutic effect in experimental acute pancreatitis

**Fig. 8:**
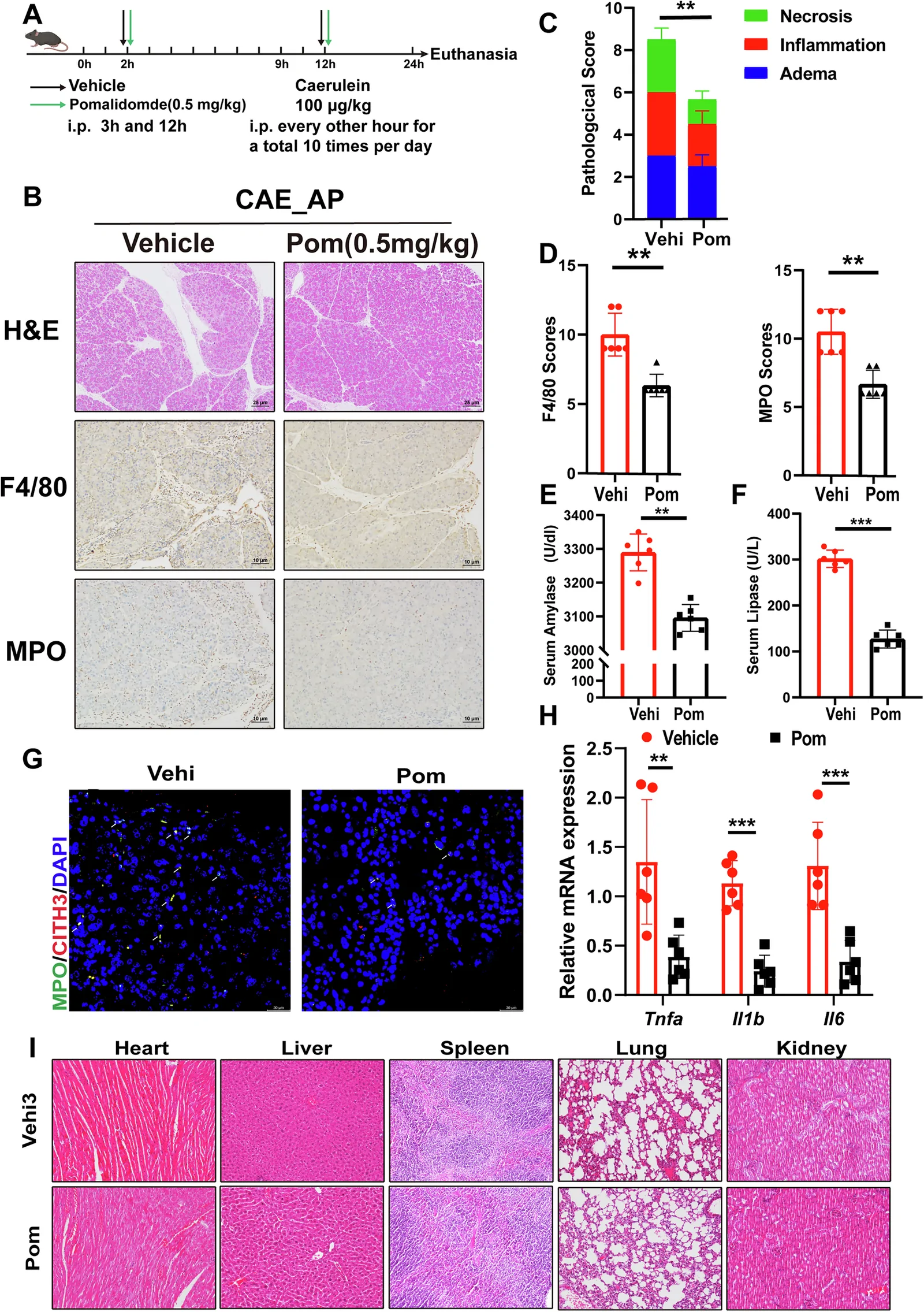
TNFα blockade after acute pancreatitis onset attenuates pancreatic injury. **A** Experimental timeline of caerulein-induced AP followed by intraperitoneal pomalidomide treatment (0.5 mg/kg i.p.). **B** Representative H&E-stained pancreatic sections from pomalidomide-treated and vehicle control mice. **C** Histopathological scoring of pancreatic injury severity (edema/necrosis/inflammation). **D** IHC scoring of MPO (left) and F4/80 (right) infiltration. **E**, **F** Serum amylase and lipase levels post-treatment. **G** IF co-staining of MPO (green) and CitH3 (red) demonstrating less NET formation. **H** qRT-PCR validation of suppressed pancreatic inflammatory cytokines (*Tnfα*, *Il-1β*, *Il-6*). **I** H&E staining of different organ between group Vehicle and group Pom. *P < 0.05, **P < 0.01, and ***P < 0.001 were considered significant. Data were expressed as the means ± SD.

To further evaluate the therapeutic potential of pomalidomide in experimental acute pancreatitis, we tested a modified treatment protocol involving intraperitoneal administration of pomalidomide at 3 and 12 h after AP induction (Fig. 8A). Consistent with our prophylactic findings, therapeutic administration of pomalidomide showed robust efficacy in ameliorating AP severity, significantly attenuating pancreatic inflammation and necrosis as shown by histopathological assessment (Fig. 8B, C) and reducing neutrophil and macrophage infiltration detected by IHC staining for MPO and F4/80 (Fig. 8D). Moreover, qRT-PCR analysis confirmed marked downregulation of pro-inflammatory factors (*TNFα*, *Il-6*, *Il-1β*) in pancreatic tissue (Fig. 8H), accompanied by reduced serum amylase and lipase levels (Fig. 8E, F). Notably, pomalidomide treatment significantly impaired NET formation (Fig. 8G). Furthermore, H&E staining revealed no detectable toxicity in other organs, including the heart, liver, spleen, lung, and kidney, of pomalidomide‑treated experimental AP mice (Fig. 8I). These results conclusively demonstrate that pomalidomide, through pharmacological inhibition of the TNF/TNFR1 axis, achieves robust therapeutic effects in mouse experimental AP.

## Discussion

The TNF/TNFR1 axis plays a pivotal role in inflammatory diseases, yet its function in AP pathogenesis remains poorly understood. The TNF/TNFR1 axis was found driving inflammation and apoptosis in various diseases. It mediates acute and chronic itch via peripheral and central mechanisms, as *Tnfr1*^–/–^ mice show reduced scratching-highlighting its therapeutic potential for pruritus [26]. *Tnfr1* deficiency reduces keratinocyte apoptosis and inflammation without impairing epidermal differentiation, marking it as a dermatological target [27]. It also suppresses melanoma by modulating tumor cell behavior and CD8^+^ T cell responses, highlighting the antitumor potential of disrupting this axis [28]. Our study bridges this critical knowledge gap by demonstrating that the TNF/TNFR1 axis was consistently upregulated across three distinct AP models. Genetic disruption of this axis markedly attenuated pancreatic inflammation. Moreover, pharmacological inhibition with pomalidomide—administered either before or after AP induction—dramatically ameliorated disease severity, suppressed inflammation, reduced NET formation, and alleviated acinar cell necrosis. These findings establish the TNF/TNFR1 axis as a key driver of AP pathogenesis.

Igkc encodes the constant region of the immunoglobulin κ light chain. Under physiological conditions, *Igkc* is primarily expressed in B lymphocytes and plasma cells, and it forms complete antibody molecules (IgG, IgM, etc.), mediating humoral immune responses, neutralizing pathogens, or opsonizing antigens for immune clearance. In our study, *Tnfr1* ablation gave rise to an inflammation-suppressive myeloid subcluster with high Igkc expression. Recent studies have shown that *Igkc* is not restricted to B lymphocytes and plasma cells. For instance, various epithelial‑derived tumor cells can synthesize Igkc; overexpression of Igkc or full‑length tumor‑derived IgG activates survival signaling pathways (e.g., PI3K/Akt, MAPK), promotes breast cancer cell proliferation, and suppresses chemotherapy‑ or radiation‑induced apoptosis [29–31]. Interestingly, in esophageal squamous cell carcinoma, a cancer‑associated fibroblast subcluster with high *Igkc* expression also exhibits inflammation-suppressive functions [32], whereas in pancreatic ductal adenocarcinoma, *Igkc*^high^ fibroblasts have not been functionally characterised [33]. Similarly, in myeloid cells, an *Igkc*^high^ macrophage subset has been reported in atherosclerosis [34], and in breast cancer, an *Igkc*^high^ myeloid subcluster is closely associated with the immunoreactivity status during therapy [35]. We will continue to investigate this area and explore the specific mechanisms by which *Igkc* functions in myeloid cells.

Marcksl1 is encoded by the *Marcksl1* gene, which is primarily involved in actin dynamics, plasma membrane maintenance, and signal transduction [36, 37]. For example, Marcksl1 controls blood vessel shape and size by modulating the mechanical response of endothelial cells to hemodynamic forces [38]. Studies have shown that Marcksl1 is closely linked to inflammation, as it promotes inflammation-driven cell migration and cytokine secretion [39]. A similar study demonstrated that autocrine TNFα signaling induces high Marcksl1 expression in macrophages, recruiting and activating immune cells and thereby contributing to peripheral neuritis [40]. Based on these observations, we speculate that the markedly reduced *Marcksl1* expression in neutrophils following *Tnfr1* knockdown impairs neutrophil migration, thereby alleviating pancreatic inflammation. This may represent a potential therapeutic target for acute pancreatitis.

Given the current lack of targeted drug therapies for AP, clinical management remains largely supportive, with no approved immunomodulatory treatments that can stop disease progression or lower mortality. This gap highlights the urgent need for therapies that address the underlying disease mechanisms. In our search for effective inhibitors of the TNF/TNFR1 axis, we systematically evaluated three distinct compounds: infliximab, necrostatin-1, and pomalidomide. Infliximab, a clinically established TNFα-neutralizing antibody, has revealed efficacy in treating inflammatory bowel disease and autoimmune hepatitis [41, 42], though its organ toxicity remains a significant clinical concern [43]. Necrostatin-1, while not directly interfering with TNF/TNFR1 axis per se, acts downstream by inhibiting receptor-interacting protein kinase 1 to block TNFα-induced necroptosis [44]. Pomalidomide, a clinically approved chemotherapeutic agent typically combined with dexamethasone for multiple myeloma treatment [45, 46], operates through a unique mechanism by enhancing mRNA degradation enzymes (e.g., Ikaros/Aiolos) to suppress TNFα transcription and secretion. Our experimental data revealed pomalidomide as the most potent therapeutic candidate for AP. Pomalidomide offers several advantages: it can be taken orally, has a well-established safety record from its use in multiple myeloma, and works by directly suppressing TNFα production through enhanced degradation of its mRNA. While immunosuppressants find broad clinical applications, their use in AP has been largely restricted to autoimmune pancreatitis (AIP), where oral corticosteroids remain the standard treatment and rituximab is reserved for recurrent AIP cases [47]. Our study provides a potential therapeutic target and a robust experimental foundation for immunomodulatory intervention in AP, although rigorous monitoring of potential off-target organ toxicity in long-term clinical studies remains essential for translational application.

Building upon the observed elevation of the TNF/TNFR1 axis in AP, our multi-model study establishes its critical role in driving inflammatory responses, demonstrating that *Tnfr1* knockout significantly attenuates pancreatic inflammation in murine AP. Through integrated single-cell and bulk transcriptomic analyses, we uncovered a marked downregulation of interferon-related genes following *Tnfr1* ablation, accompanied by the emergence of unique inflammation-suppressive neutrophil and myeloid cell subclusters. Most notably, we identified pomalidomide as a potent therapeutic agent that not only ameliorates inflammation but also protects against acinar cell death. Our work systematically delineates the TNF/TNFR1 axis as a master regulator of AP pathogenesis, revealing its dual impact on both gene expression programs and immune cell polarization. The discovery of pomalidomide’s efficacy in AP management represents a conceptual advance, opening new therapeutic insight and suggesting promising immunomodulatory strategies for clinical translation.

### Limitations of the study

While this study presents significant novel findings, several limitations warrant discussion. First, we have not fully elucidated the molecular mechanisms of the TNF/TNFR1 axis signaling, particularly regarding its upstream transcriptional regulators and downstream effectors. Second, although we identified differentially expressed genes in neutrophils and macrophages that likely interact with the TNF/TNFR1 axis to modulate AP progression, these candidates remain to be functionally validated through pharmacological interventions (inhibitors/activators) in AP models. Furthermore, our scRNA-seq analysis requires deeper exploration, particularly concerning acinar cell alterations and functional characterization of the newly identified immune cell subsets. These limitations directly inform our future research directions: we will systematically investigate the downstream molecular consequences of *Tnfr1* ablation, delineate its impact on acinar cell biology, and experimentally validate the candidate genes identified through multi-omics analysis to assess their effects on TNF/TNFR1 axis and AP severity. Collectively, these efforts aim to establish a comprehensive TNF/TNFR1-centric regulatory pathway governing pancreatic inflammation, potentially revealing novel therapeutic strategies for AP management.

## Supplementary information

Supplementary Figure S1-S7, Supplementary File S1-S2, Supplementary Table S1-S3

Table S4

Table S5

Table S6

Table S7

Table S8

Table S9

Table S10

Table S11

Table S12

## Data Availability

The single-cell transcriptomic and bulk RNA-seq data of pancreatic tissues from acute pancreatitis have been deposited in the Gene Expression Omnibus (GEO) database under accession numbers GSE309230 and GSE306650. In addition, this study analyzed publicly available scRNA-seq data from GEO (GSE188819).
