# Supplementary material for: TNF/TNFR1 axis restrains inflammation-suppressive myeloid subclusters to drive acute pancreatitis
Source: Cell Death Dis. 2026 Aug 26;17(1):906. doi: 10.1038/s41419-026-09208-9 (PMC13694277; doi:10.1038/s41419-026-09208-9)
Supplement: Supplementary file 1 [file 41419_2026_9208_MOESM1_ESM.pdf]

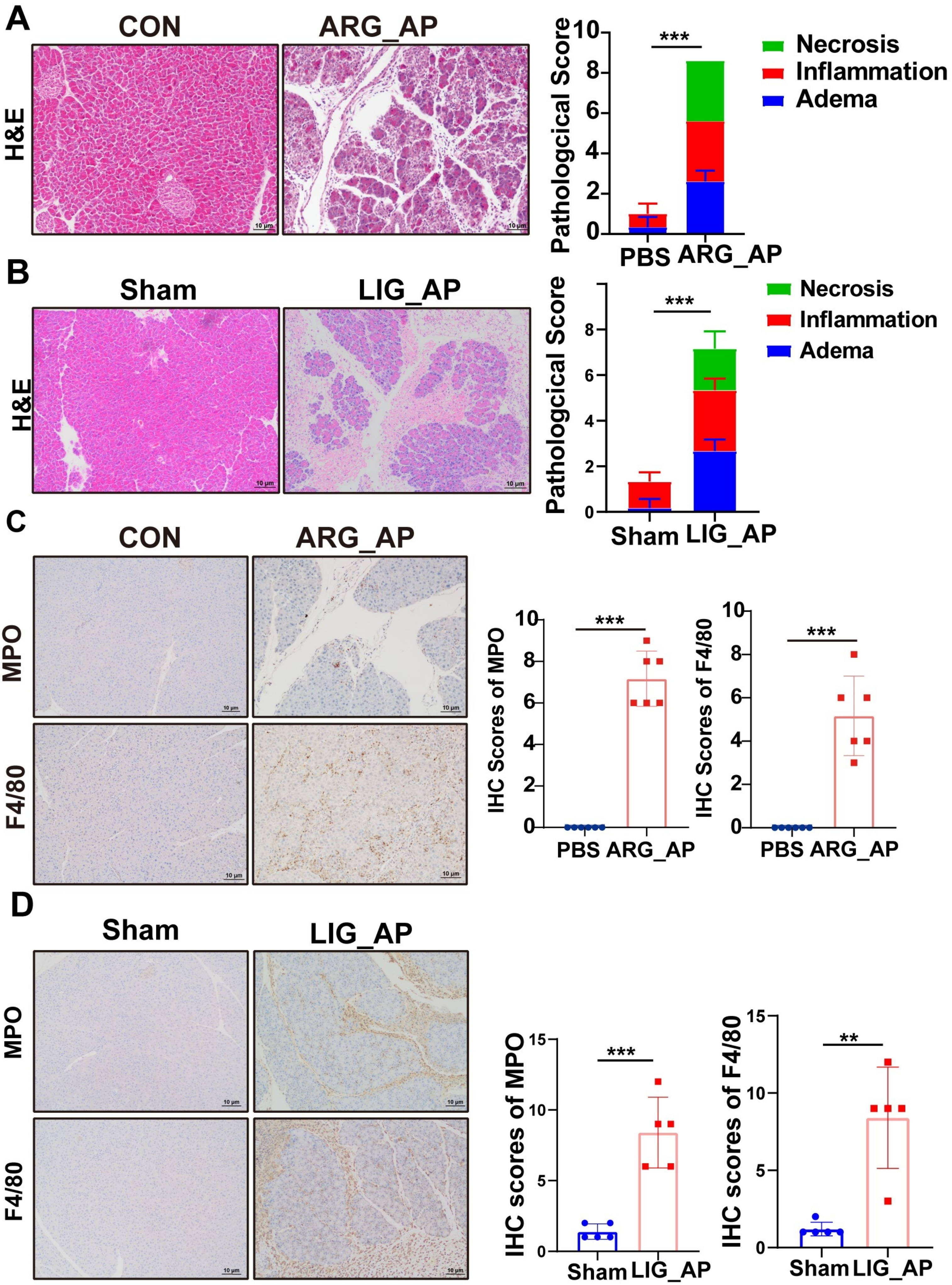

A
CON
ARG_AP
H&E
Pathologcical Score
***
Necrosis
Inflammation
Adema
PBS
ARG_AP
B
Sham
LIG_AP
H&E
Pathologcical Score
***
Necrosis
Inflammation
Adema
Sham
LIG_AP
C
CON
ARG_AP
MPO
F4/80
IHC Scores of MPO
IHC Scores of F4/80
***
***
PBS
ARG_AP
PBS
ARG_AP
D
Sham
LIG_AP
MPO
F4/80
IHC scores of MPO
IHC scores of F4/80
***
**
Sham
LIG_AP
Sham
LIG_AP

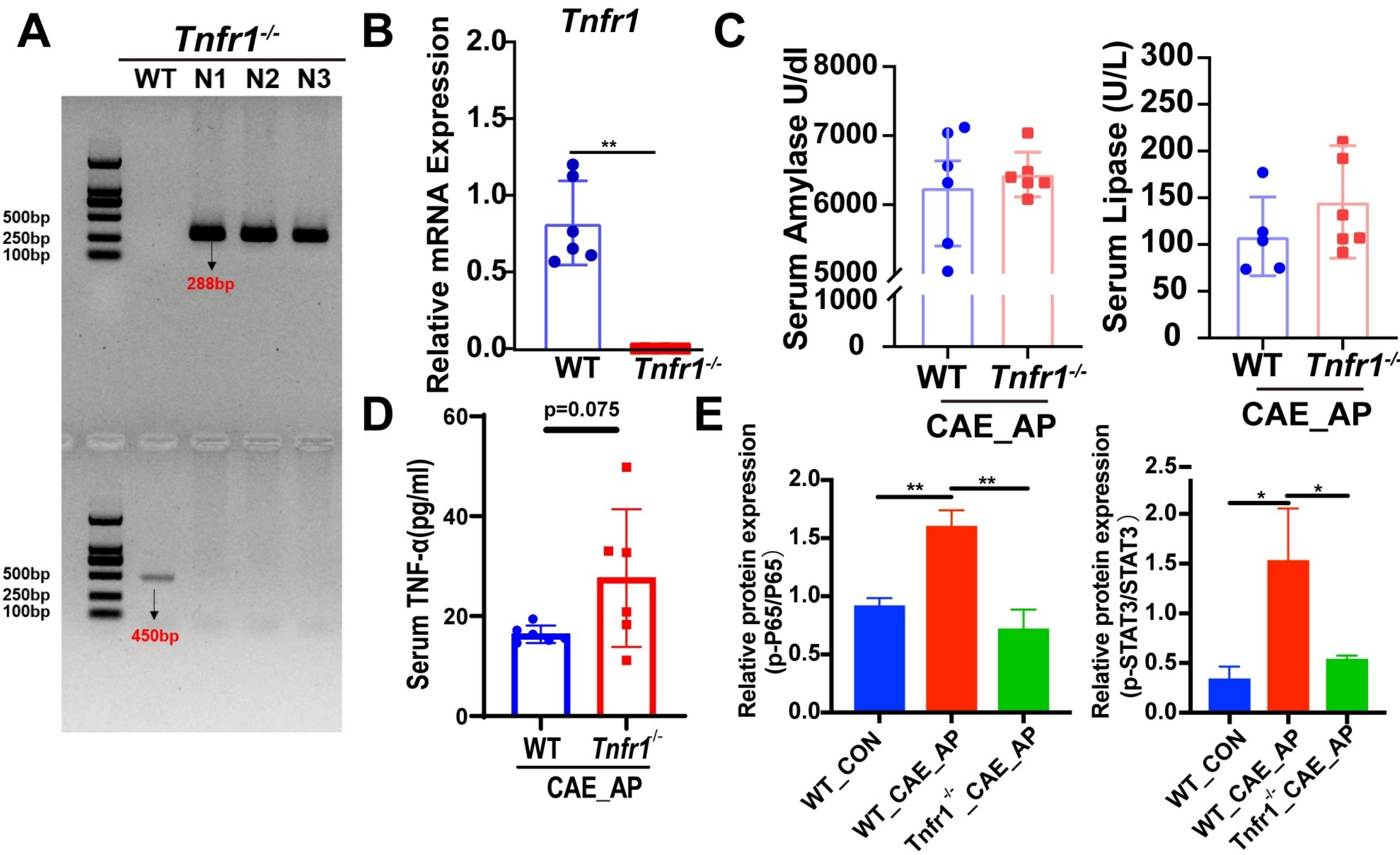

A
Tnfr1-/-
WT N1 N2 N3
500bp
250bp
100bp
288bp
500bp
250bp
100bp
450bp
B
Tnfr1
Relative mRNA Expression
**
WT Tnfr1-/-
C
Serum Amylase U/dl
WT Tnfr1-/-
CAE_AP
Serum Lipase (U/L)
WT Tnfr1-/-
CAE_AP
D
Serum TNF-α(pg/ml)
p=0.075
WT Tnfr1-/-
CAE_AP
E
Relative protein expression (p-P65/P65)
** **
WT_CON WT_CAE_AP Tnfr1-/-_CAE_AP
Relative protein expression (p-STAT3/STAT3)
* *
WT_CON WT_CAE_AP Tnfr1-/-_CAE_AP

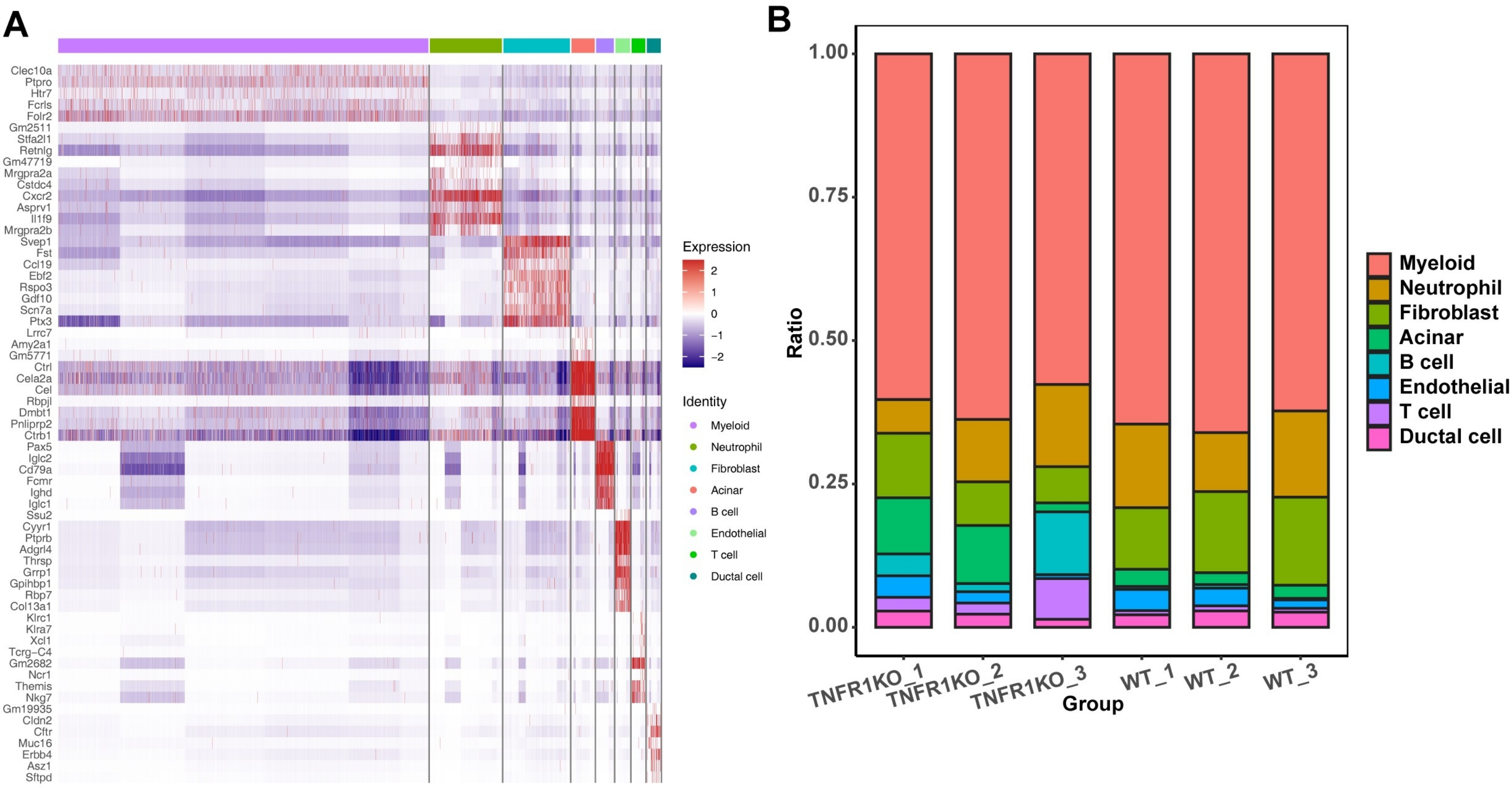
A
Clec10a
Ptpro
Htr7
Fcrls
Folr2
Gm2511
Stfa2l1
Retnlg
Gm47719
Mrgpra2a
Cstdc4
Cxcr2
Asprv1
Il1f9
Mrgpra2b
Svep1
Fst
Ccl19
Ebf2
Rspo3
Gdf10
Scn7a
Ptx3
Lrrc7
Amy2a1
Gm5771
Ctrl
Cela2a
Cel
Rbpjl
Dmbt1
Pnliprp2
Ctrb1
Pax5
Iglc2
Cd79a
Fcmr
Ighd
Iglc1
Ssu2
Cyyr1
Ptprb
Adgrl4
Thrsp
Grrp1
Gpihbp1
Rbp7
Col13a1
Klrc1
Klra7
Xcl1
Tcrg−C4
Gm2682
Ncr1
Themis
Nkg7
Gm19935
Cldn2
Cftr
Muc16
Erbb4
Asz1
Sftpd
Expression
2
1
0
−1
−2
Identity
Myeloid
Neutrophil
Fibroblast
Acinar
B cell
Endothelial
T cell
Ductal cell
B
Ratio
1.00
0.75
0.50
0.25
0.00
TNFR1KO_1
TNFR1KO_2
TNFR1KO_3
WT_1
WT_2
WT_3
Group
Myeloid
Neutrophil
Fibroblast
Acinar
B cell
Endothelial
T cell
Ductal cell

A

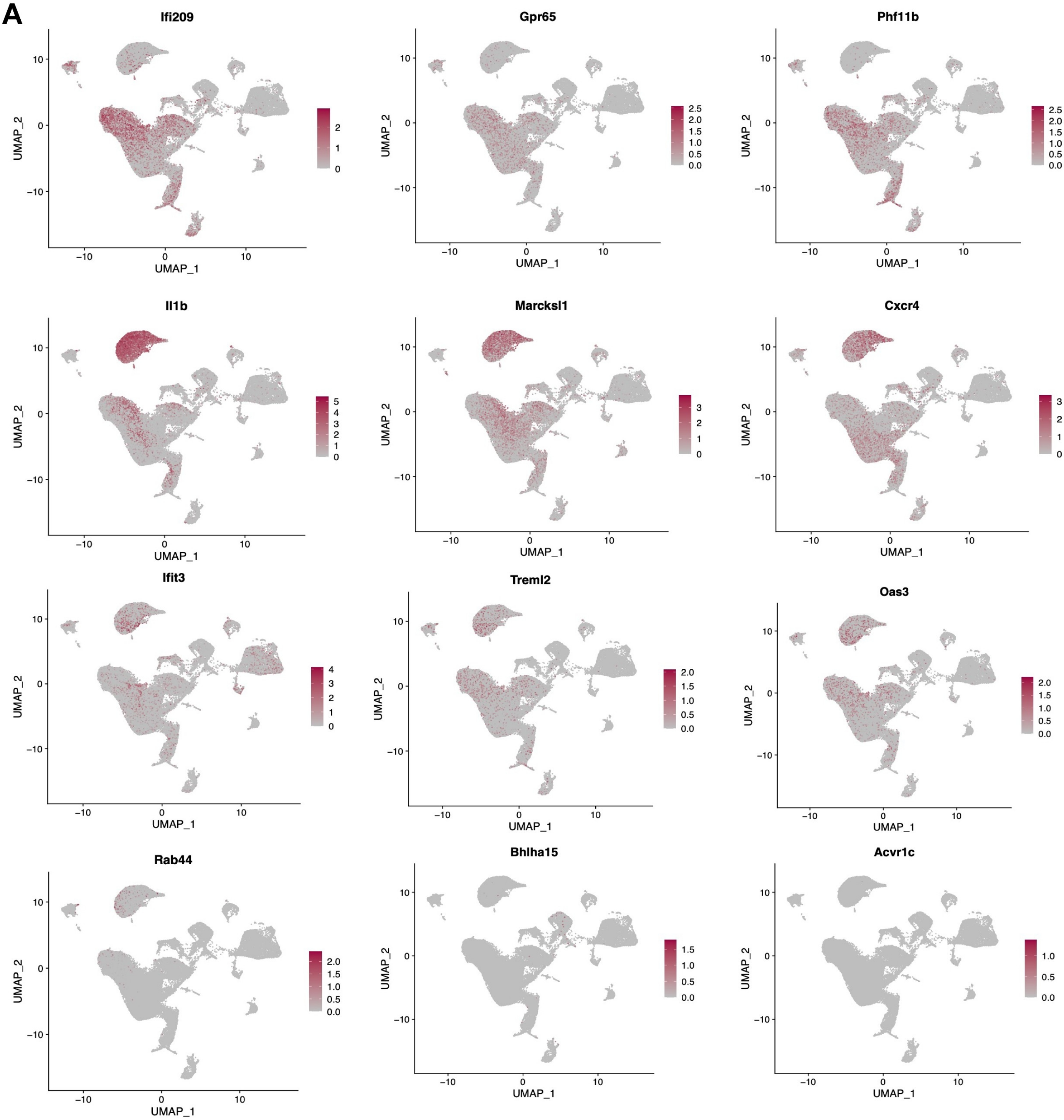

Ifi209
Gpr65
Phf11b
Il1b
Marcksl1
Cxcr4
Ifit3
Treml2
Oas3
Rab44
Bhlha15
Acvr1c
UMAP_1
UMAP_2

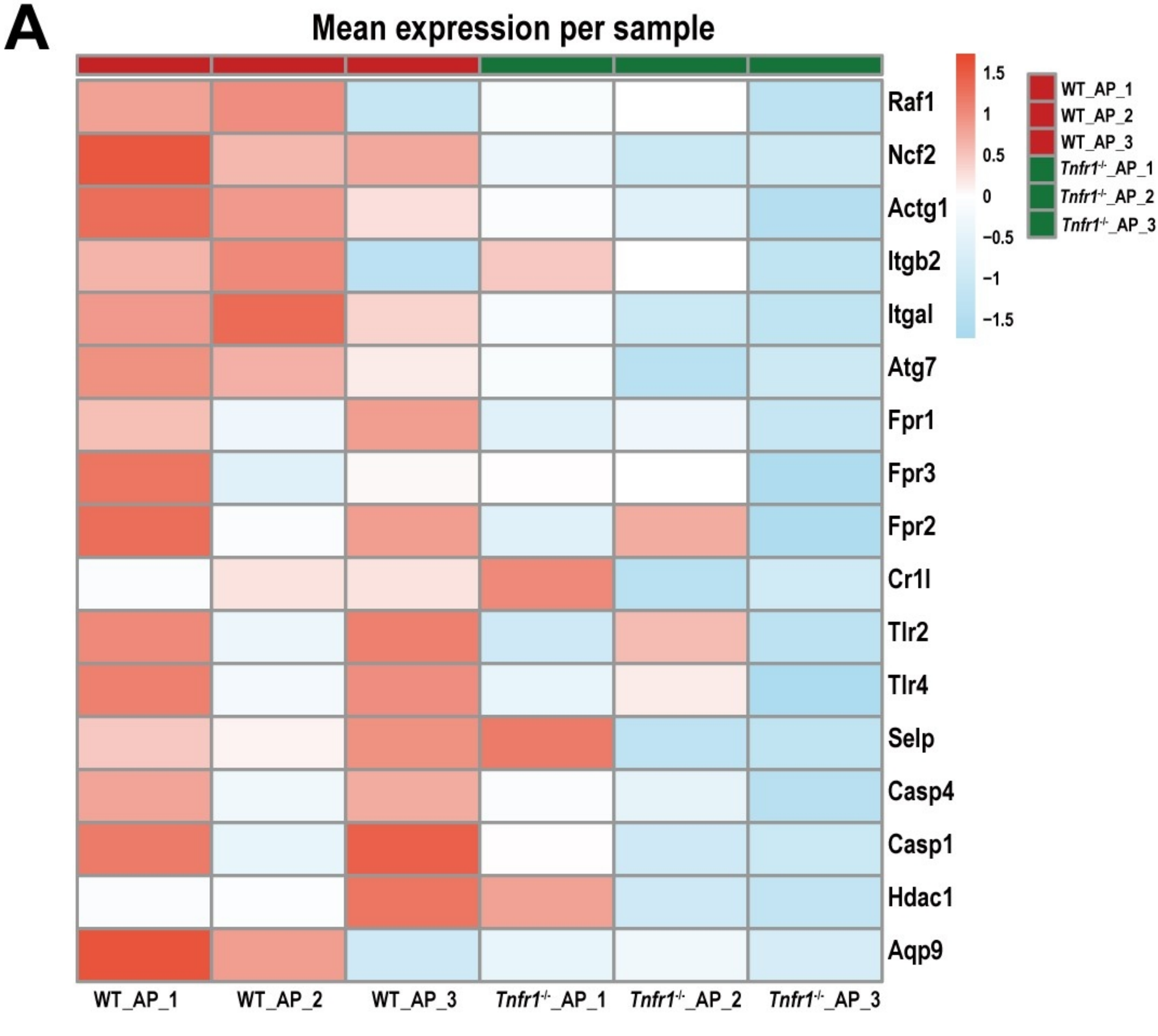

A
Mean expression per sample
Raf1
Ncf2
Actg1
Itgb2
Itgal
Atg7
Fpr1
Fpr3
Fpr2
Cr1l
Tlr2
Tlr4
Selp
Casp4
Casp1
Hdac1
Aqp9
1.5
1
0.5
0
−0.5
−1
−1.5
WT_AP_1
WT_AP_2
WT_AP_3
Tnfr1-/-_AP_1
Tnfr1-/-_AP_2
Tnfr1-/-_AP_3

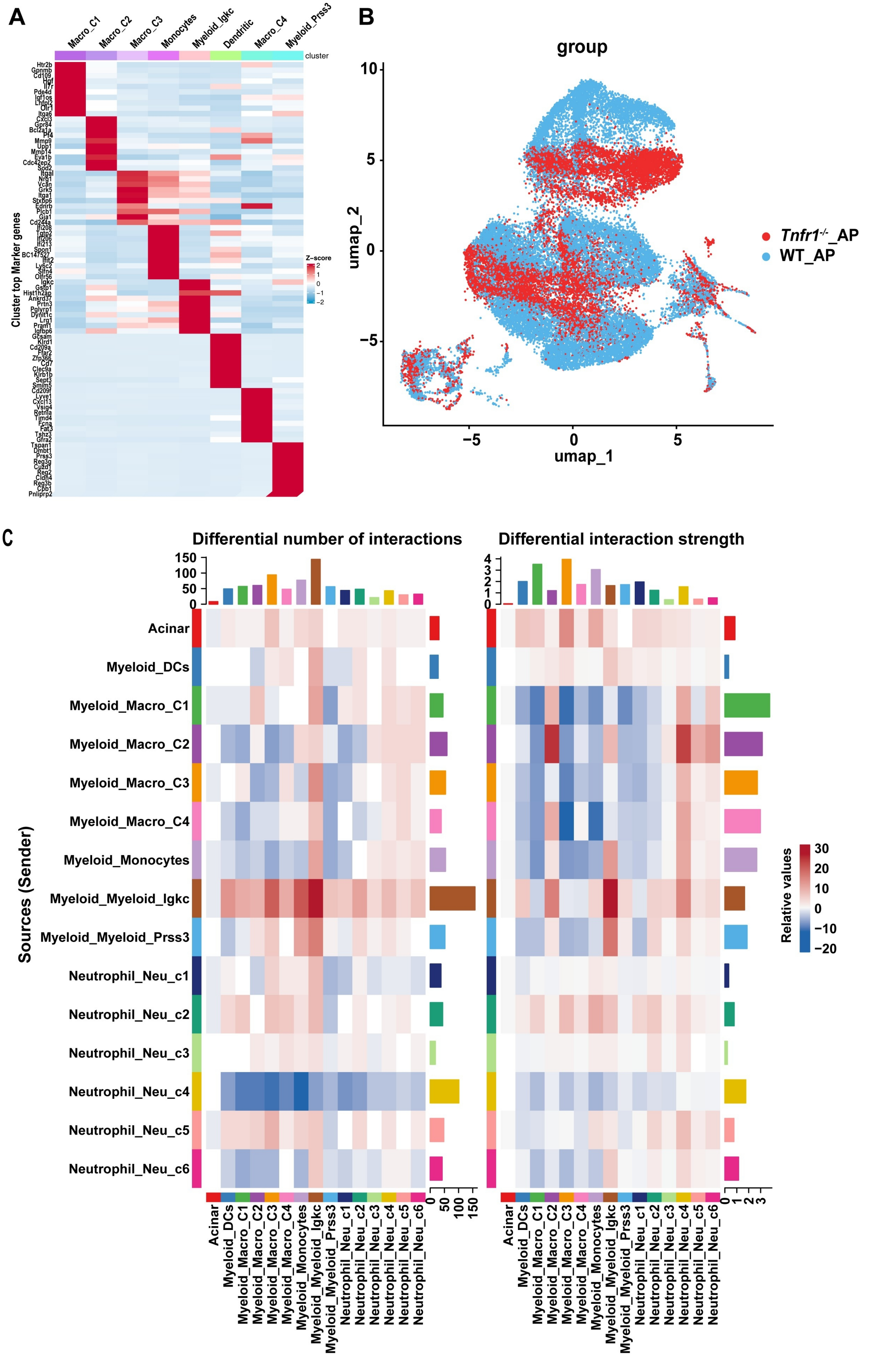
A
Macro_C1
Macro_C2
Macro_C3
Monocytes
Myeloid_Igkc
Dendritic
Macro_C4
Myeloid_Prss3
cluster
Cluster top Marker genes
Z-score
B
group
umap_2
umap_1
Tnfr1-/-_AP
WT_AP
C
Differential number of interactions
Differential interaction strength
Sources (Sender)
Relative values
Acinar
Myeloid_DCs
Myeloid_Macro_C1
Myeloid_Macro_C2
Myeloid_Macro_C3
Myeloid_Macro_C4
Myeloid_Monocytes
Myeloid_Myeloid_Igkc
Myeloid_Myeloid_Prss3
Neutrophil_Neu_c1
Neutrophil_Neu_c2
Neutrophil_Neu_c3
Neutrophil_Neu_c4
Neutrophil_Neu_c5
Neutrophil_Neu_c6

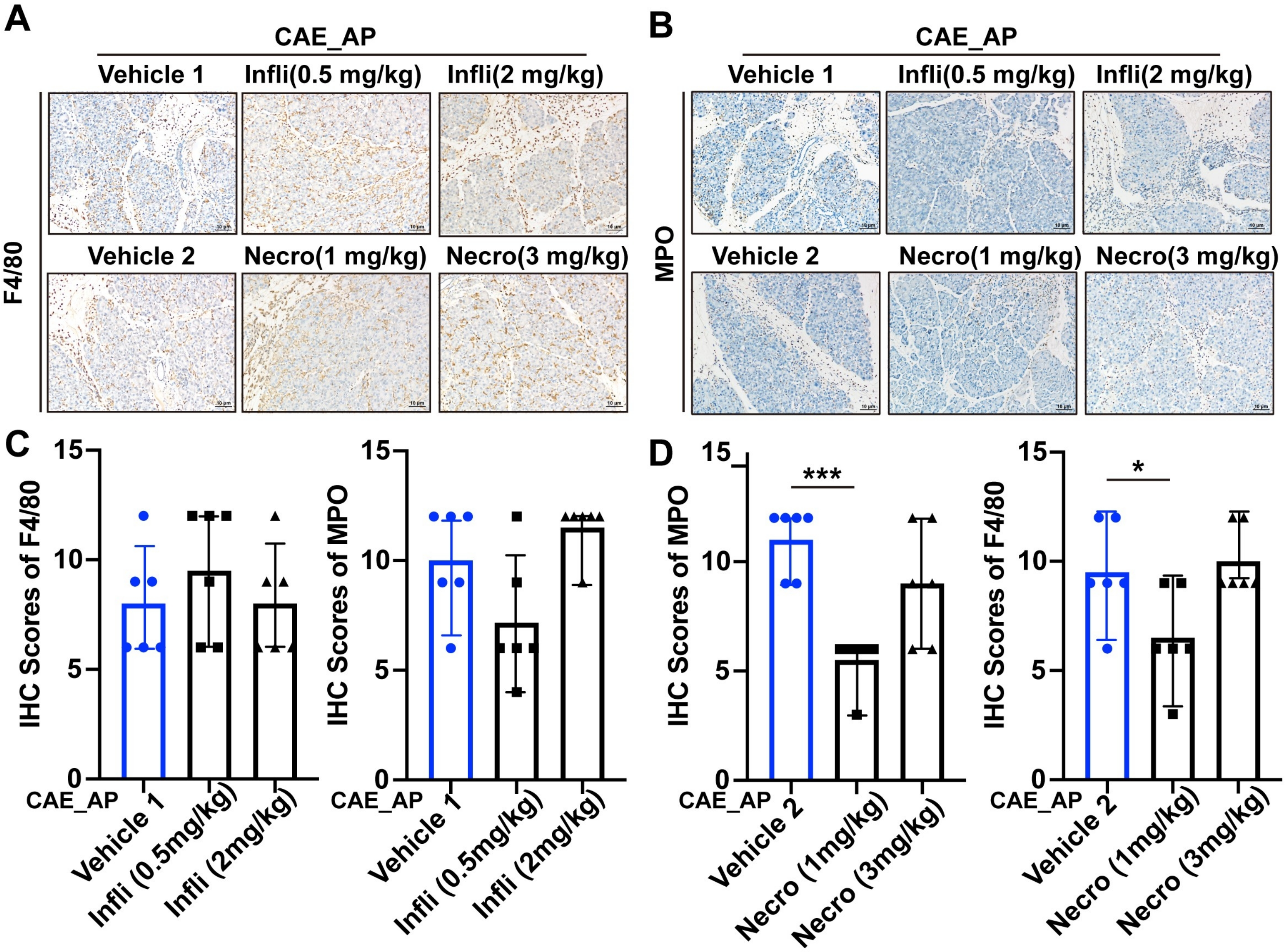
A
CAE_AP
Vehicle 1
Infli(0.5 mg/kg)
Infli(2 mg/kg)
F4/80
Vehicle 2
Necro(1 mg/kg)
Necro(3 mg/kg)
B
CAE_AP
Vehicle 1
Infli(0.5 mg/kg)
Infli(2 mg/kg)
MPO
Vehicle 2
Necro(1 mg/kg)
Necro(3 mg/kg)
C
IHC Scores of F4/80
IHC Scores of MPO
CAE_AP
Vehicle 1
Infli (0.5mg/kg)
Infli (2mg/kg)
D
IHC Scores of MPO
IHC Scores of F4/80
***
*
CAE_AP
Vehicle 2
Necro (1mg/kg)
Necro (3mg/kg)

Supplementary Figure Legends

Figure S1. Robust activation of the TNF/TNFR1 axis during experimental AP progression. (A) Representative H&E staining of pancreatic tissue sections (scale bar: 10 μm) and histopathological scores in control and ARG_AP mice. (B) Histopathological scoring of H&E staining of pancreatic tissues from sham-operated and LIG_AP mice. (C) IHC staining and quantitative analysis of MPO and macrophage F4/80 infiltration in pancreatic tissues from control and ARG_AP mice. (D) IHC staining and scores of MPO and F4/80 in pancreatic tissues from sham-operated and LIG_AP mice. (E) Western blot quantification of TNFR1 protein expression in Figure 1J. $^{*}P < 0.05$, $^{**}P < 0.01$ and $^{***}P < 0.001$ were considered significant. Data were expressed as the means ± SD.

Figure S2. Supporting data for Figure 2. (A) Genotyping of WT and *Tnfr1*$^{-/-}$ mice by tail DNA extraction and agarose gel electrophoresis. (B) qRT-PCR analysis confirming *Tnfr1* transcriptional silencing in knockout mice compared to WT controls. (C) Serum lipase and amylase levels in WT and *Tnfr1*$^{-/-}$ mice following caerulein-induced AP. (D) Serum TNFα levels in WT and *Tnfr1*$^{-/-}$ mice in AP. (E) Quantitative analysis of P65 and STAT3 phosphorylation levels from western blot data shown in Figure 2F, normalized to total protein expression. $^{*}P < 0.05$, $^{**}P < 0.01$ and $^{***}P < 0.001$ were considered significant. Data were expressed as the means ± SD.

Figure S3. Supporting data for Figure 3. (A) Heatmap displaying top marker genes for the 8 identified cell types from scRNA-seq analysis. (B) Stacked bar plot showing relative proportions of each cell type across individual mouse pancreatic samples. $^{*}P < 0.05$, $^{**}P < 0.01$ and $^{***}P < 0.001$ were considered significant. Data were expressed as the means ± SD.

Figure S4. Supporting data for Figure 4. (A) UMAP of consensus DEGs from scRNA-seq and bulk RNA-seq: *Ifi209*, *Gpr65*, *Phf11b*, *Marcksl1*, *Cxcr4*, *Ifit3*, *Treml2*,

Oas3, *Rab44*, *Bhlha15*, and *Acvr1c*.

Figure S5. Supporting data for Figure 5. (A) Heatmap showing expression levels of NET-related genes in the two groups (WT vs *Tnfr1*$^{-/-}$) based on single-cell RNA-sequencing data.

Figure S6. Supporting data for Figure 6. (A) Heatmap displaying top 5 marker genes for the 8 identified myeloid cell types from scRNA-seq analysis. (**B**) Genotype-comparative UMAP plots (WT vs *Tnfr1*$^{-/-}$) illustrating myeloid cluster redistribution. (C) Heatmap of communication strength and communication number between acinar cells and myeloid cell subclusters as well as neutrophil subclusters.

Figure S7. Supporting data for Figure 7. (A) IHC staining of MPO infiltration in different groups; (B) IHC staining of F4/80 infiltration in different groups; (C) The IHC scores of MPO and F4/80 comparing the vehicle 1 and infliximab groups. (D) The IHC scores of MPO and F4/80 comparing the vehicle 2 and necrostatin-1 group. $*P < 0.05$, $**P < 0.01$ and $***P < 0.001$ were considered significant. Data were expressed as the means ± SD.

File S1 Original data （Raw Western blot images from this study）

Figure 1J

GAPDH

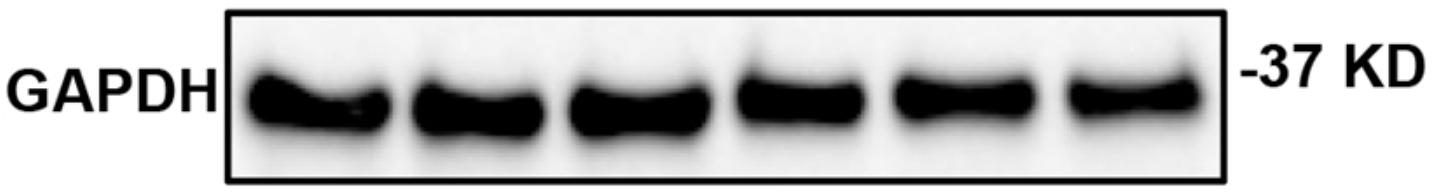

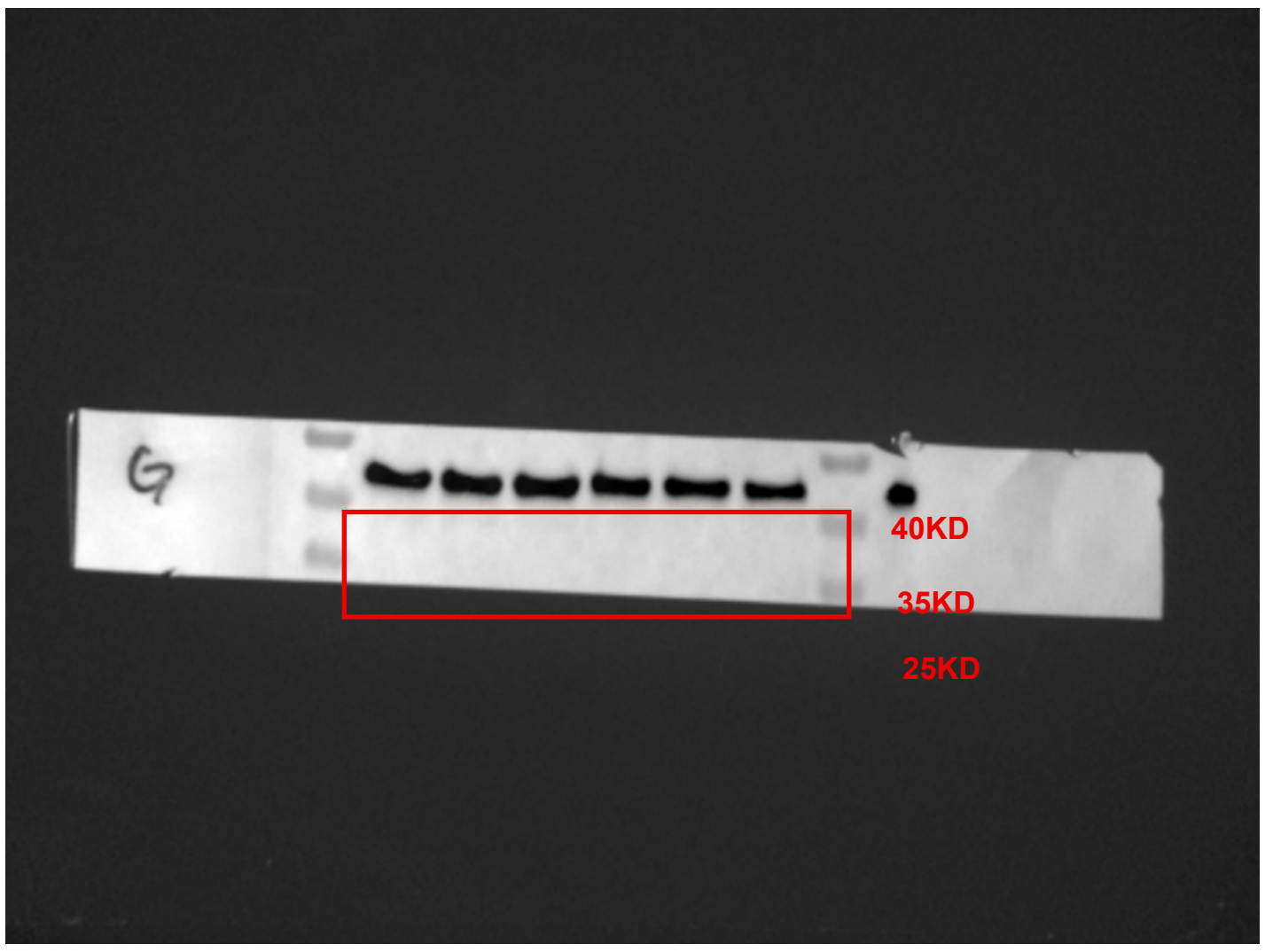

TNFR1

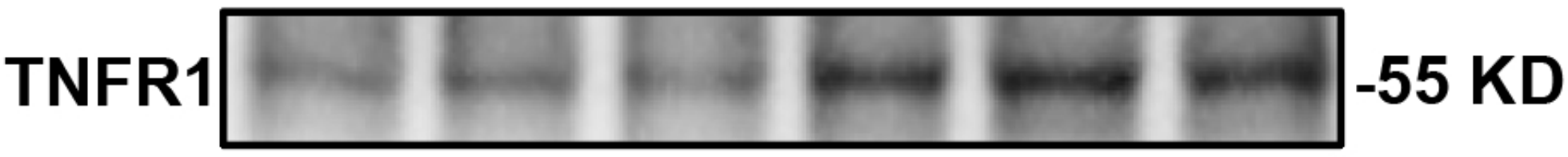

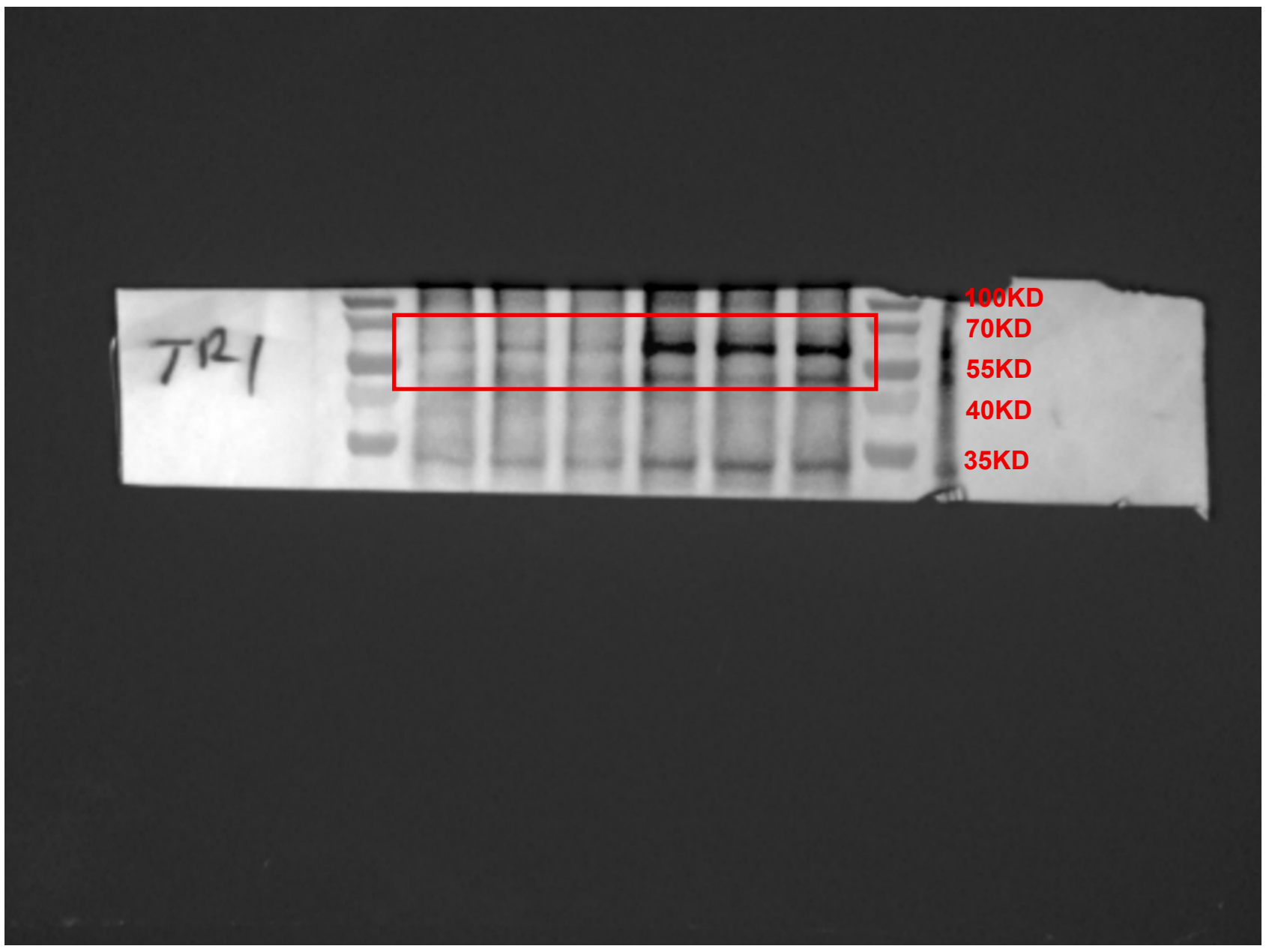

Figure 2F

GAPDH

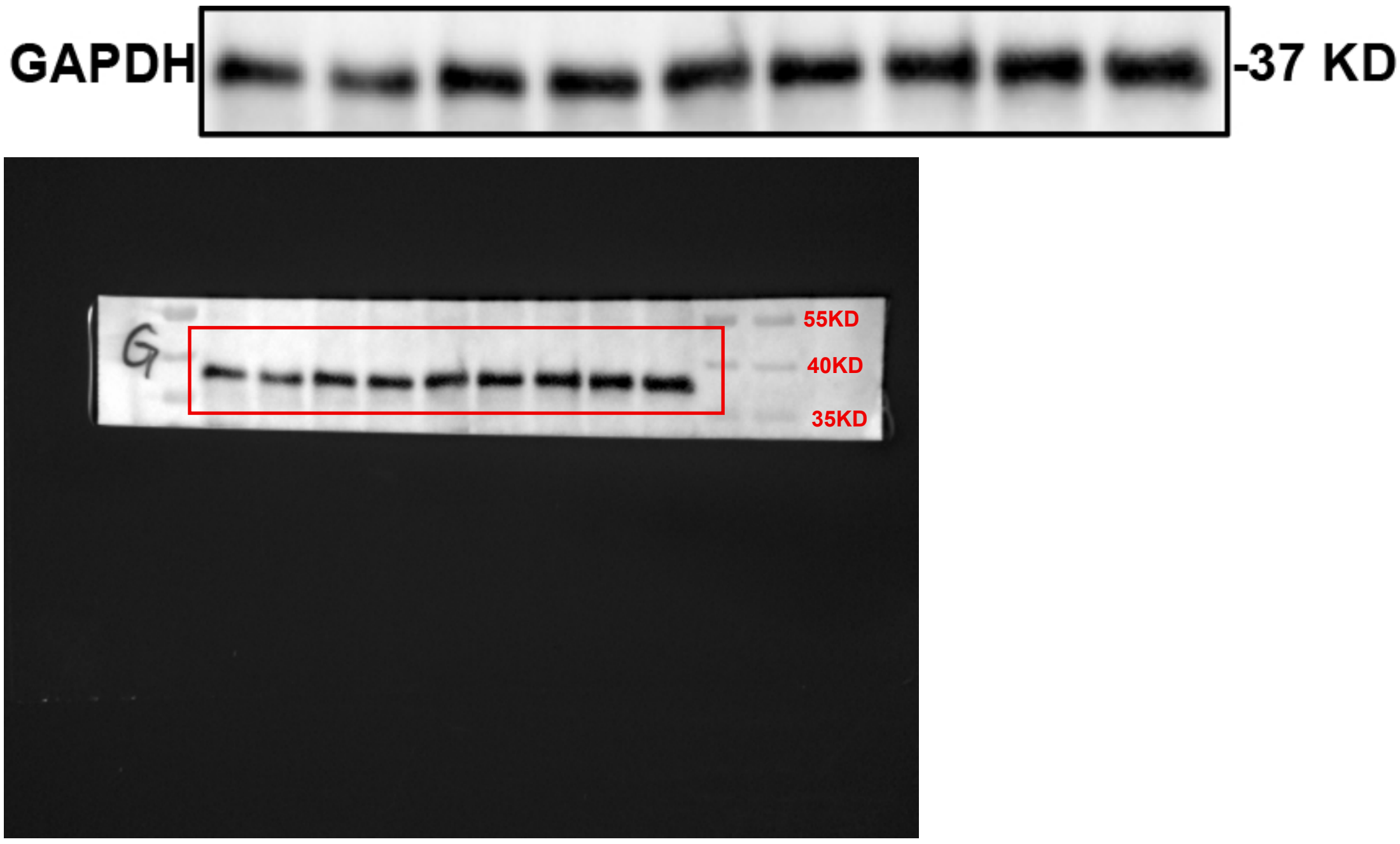

P65

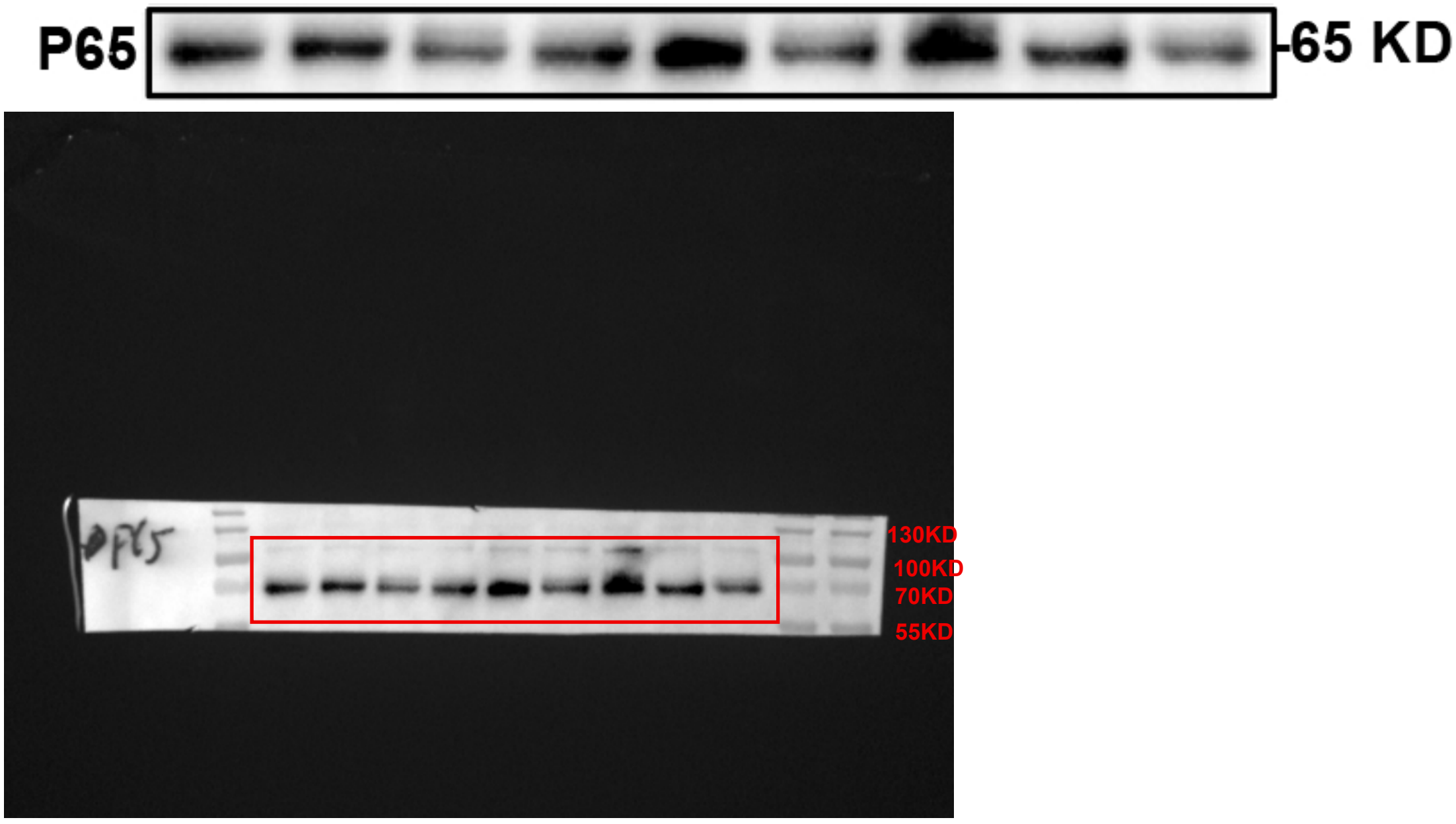

p-P65

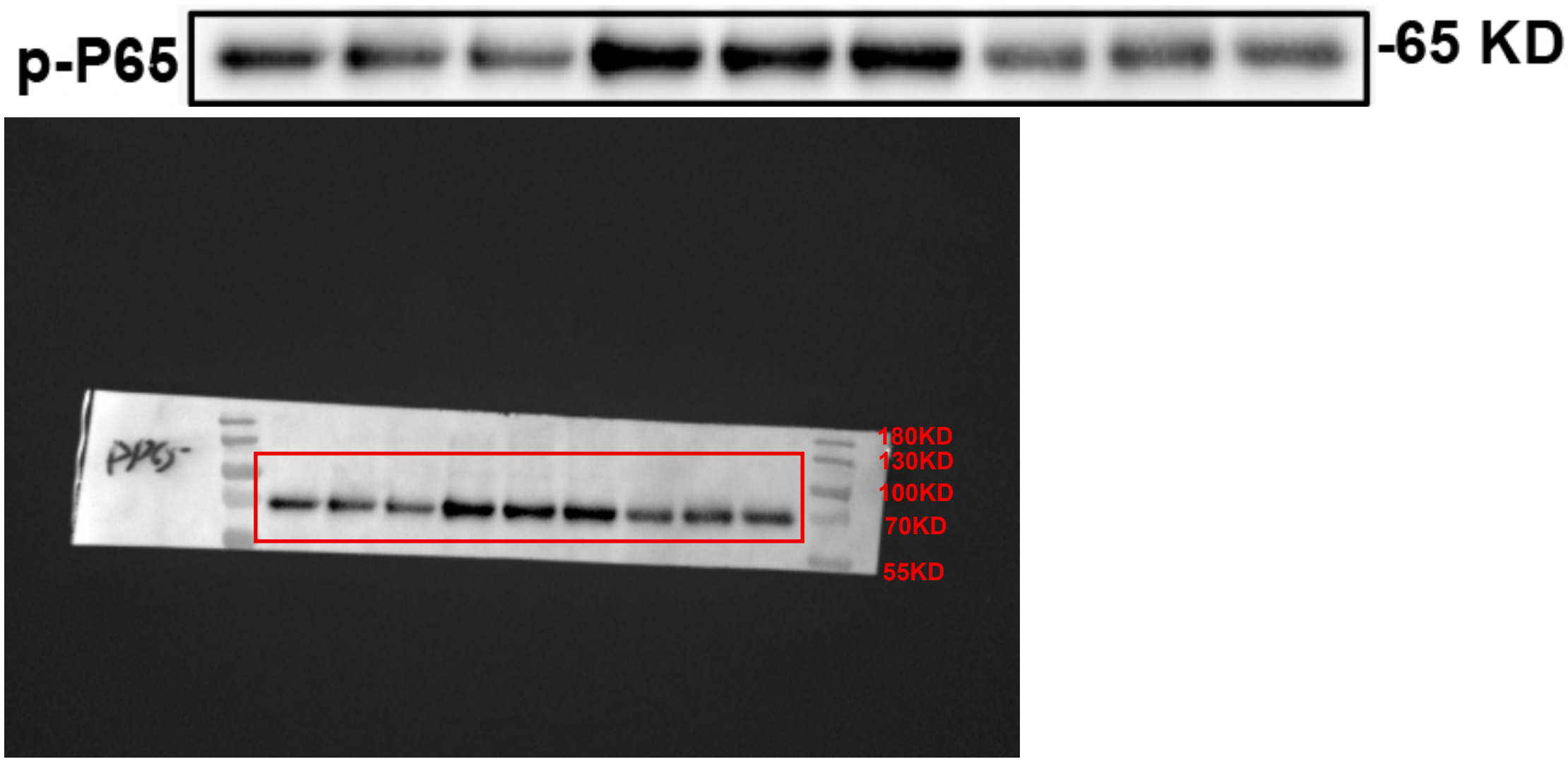

p-P65
-65 KD
180KD
130KD
100KD
70KD
55KD

p-STAT3

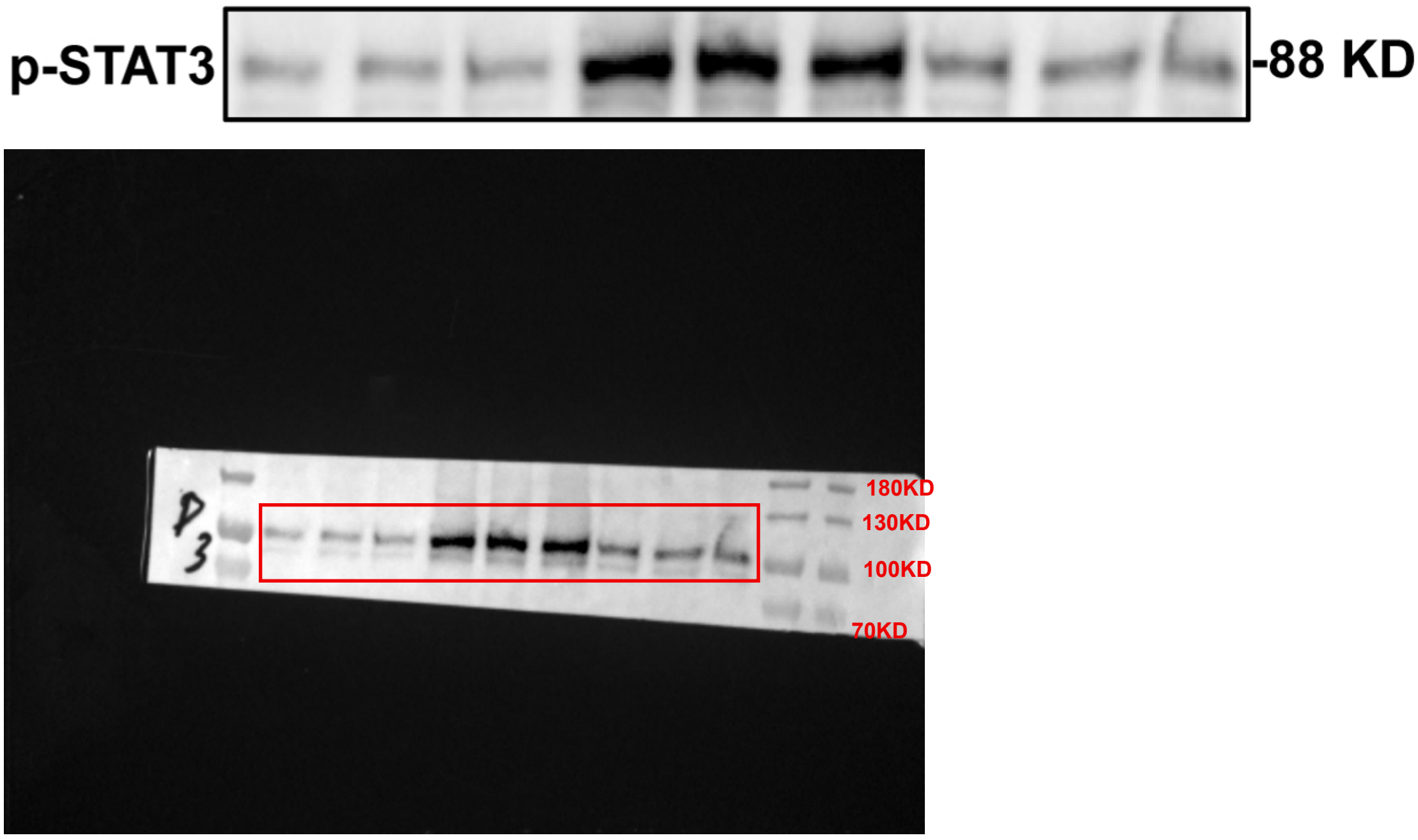

p-STAT3
-88 KD
180KD
130KD
100KD
70KD

STAT3

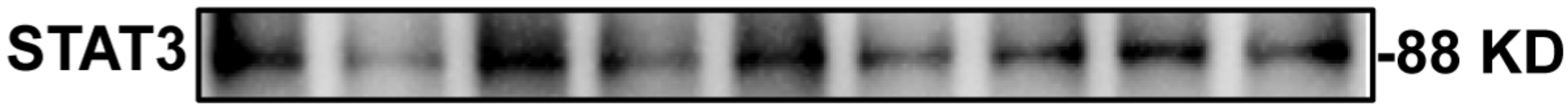

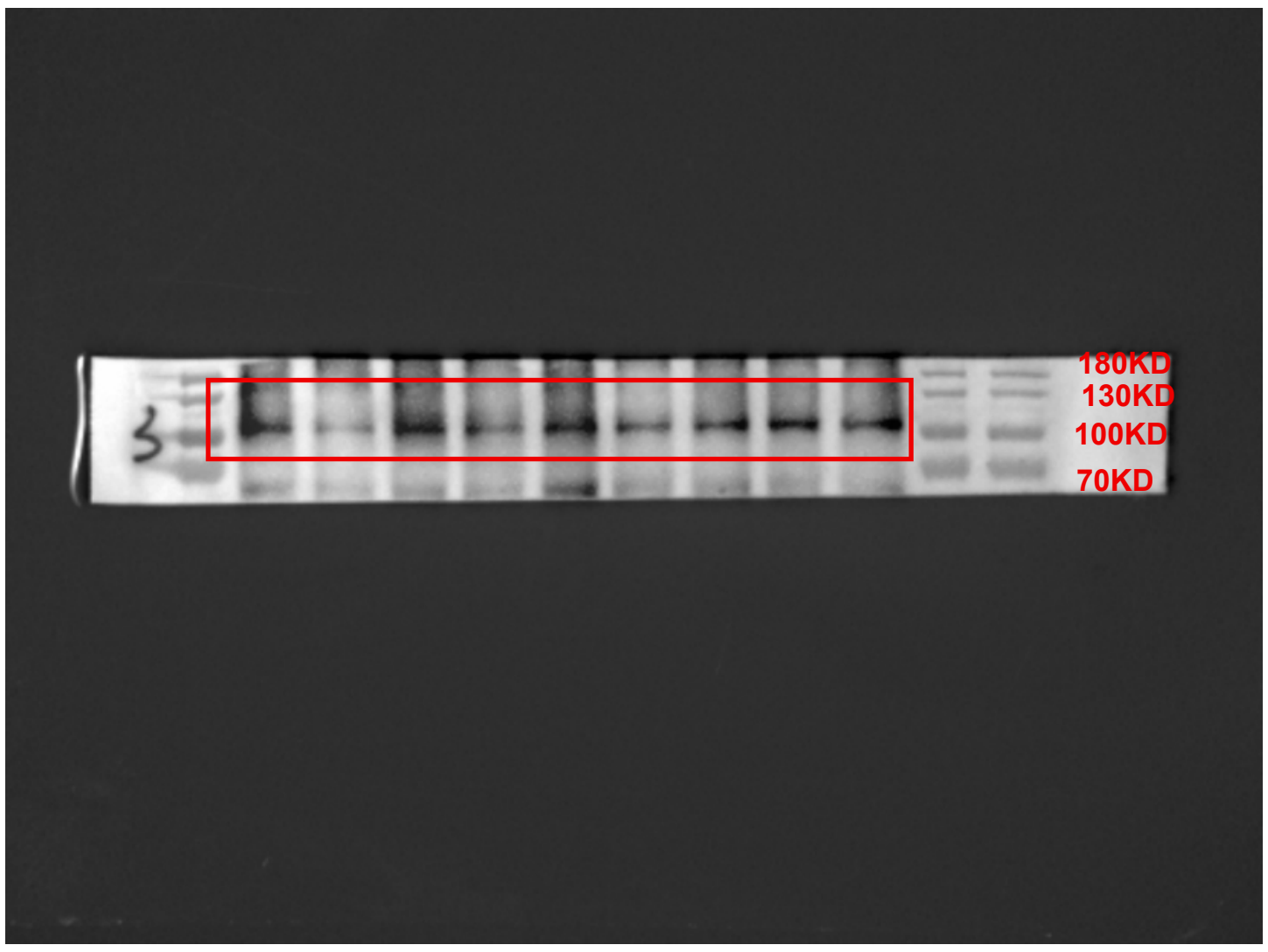

File S2 Analysis of clinical parameters and TNFTNFR1 expression in patients with AP

Variables	Crude modelOR (95%CI)	*P* value	Model 1OR (95%CI)	*P* value	Model 2OR (95%CI)	*P* value	Model 3OR (95%CI)	*P* value
TNFR1	1.22 (1.08~1.39)	0.002	1.2 (1.05~1.36)	0.007	1.2 (1.05~1.36)	0.007	1.19 (1.05~1.36)	0.007
TNFa	1.07 (1.03~1.12)	0.002	1.09 (1.02~1.17)	0.011	1.1 (1.02~1.18)	0.013	1.1 (1.02~1.2)	0.016

Model Adjustments:
Crude model: Adjusted for None.
Model 1: Adjusted for age, sex,RR,HR,Temp,MAP
Model 2: Further adjusted for WBC,HCT,Hb
Model 3: Further adjusted for ALT,TBIL,Cr,TG

Variables	MAP/MSAP (n = 44)	SAP (n = 15)	P
Age	43.7 ± 14.5	45.6 ± 12.1	0.663
Sex			0.714
Female	17 (38.6)	5 (33.3)	
male	27 (61.4)	10 (66.7)	
Respiratory rate	22.3 ± 5.1	25.7 ± 7.4	0.054
Heart rate	103.7 ± 19.5	115.0 ± 17.4	0.052
Temperature	36.9 ± 0.6	37.0 ± 0.4	0.58
Mean arterial pressure	98.6 ± 11.1	98.2 ± 14.2	0.914
TNFR1	66.3 ± 6.6	73.6 ± 5.5	< 0.001
TNFa	1021.1 ± 46.0	1137.6 ± 35.5	< 0.001
WBC	16.0 ± 5.5	14.5 ± 7.6	0.414
Hb	144.4 ± 18.3	148.5 ± 19.8	0.47
HCT	42.4 ± 5.2	44.2 ± 5.9	0.287
PLT	241.3 ± 70.4	215.0 ± 95.0	0.259
ALT	36.9 (20.0, 66.4)	37.9 (16.4, 64.4)	0.542
AST	29.6 (22.1, 67.8)	39.8 (30.0, 61.0)	0.389
TBIL	18.0 (11.3, 30.6)	15.5 (11.8, 18.5)	0.22
DBIL	5.0 (2.8, 12.7)	5.2 (2.8, 9.1)	0.875
ALB	39.4 ± 5.4	38.0 ± 4.9	0.385
TG	9.9 (2.8, 14.3)	13.2 (2.6, 24.9)	0.317
GLU	9.8 ± 5.7	14.2 ± 5.0	0.009
BUN	4.8 ± 1.6	5.6 ± 2.7	0.169
Cr	77.9 ± 30.4	96.5 ± 53.5	0.101

WBC, white blood cell; Hb, hemoglobin; HCT, hematocrit; PLT, platelet; ALT, alanine aminotransferase; AST, aspartate aminotransferase; TBIL, total bilirubin; DBIL, direct bilirubin; ALB, albumin; TG, triglyceride; GLU, glucose; BUN, blood urea nitrogen; Cr, creatinine

Supplementary Table 1. The primary antibodies used in this study				
Antibody	Source	Identifier	Application	Working dilution
Anti-Tnf-± antibody	Santa Cruze	sc-52746	IHC/IF	1:50
Anti-Tnfr1 antibody	Abcolonal	A1540	WB	1:1000
			IHC	1:50
Anti-Mpo antibody	Abcam	ab20867	IHC	1:1000
Anti-F4/80 antibody	Servicebio	GB113373-100	IHC	1:1000
Anti-Mpo antibody	R&D System	AF3667	IF	1:500
Anti-Cith3 antibody	Abcam	ab5103	IF	1:100
Anti-S100a9 antibody	abcam	ab24295	IF	1:1000
Anti-Lyz antibody	abcam	ab108508	IF	1:400
Anti-Amylase antibody	Santa Cruze	sc-46657	IF	1:100
Anti-Ly6G antibody	BioLegend	127602	IF	1:400
Anti-Marcksl1 antibody	Santa Cruze	sc-130471	IHC	1:500
Anti-P65 antibody	Proteintech	80979-1-RR	WB	1:1000
Anti-p-P65 antibody	Proteintech	82335-1-RR	WB	1:1000
Anti-Stat3 antibody	Proteintech	10253-2-AP	WB	1:1000
Anti-p-Stat3 antibody	Proteintech	60479-1-Ig	WB	1:1000
Anti-mouse antibody	NCM biotech	P8001	WB	1:2500
Anti-rabbit antibody	NCM biotech	P8002	WB	1:2500
Goat anti-Mouse IgG (H+L) Cross-Adsorbed Secondary Antibody, Alexa Fluor" 488	Invitrogen	A-11001	IF	1:500
Goat anti-Rabbit IgG (H+L) Cross-Adsorbed Secondary Antibody, Alexa Fluor" 555	Invitrogen	A-21428	IF	1:500
CD11b-PE-Cy7	BioLegend	101215	Flow Cytometry	1:50
F4/80-PE	BD Biosciences	565410	Flow Cytometry	1:50
Ly6G-PerCP-Cy5.5	BD Biosciences	560602	Flow Cytometry	1:50

Supplementary Table 2. Sequences of primers in this study			
Gene name	Species	Sequences	
		Forward	Reverse
Tnfrsf1a F1/R1	mouse	52CTTCCTTTTGTTTCTGTCCCG-32	52TCTAAAGCAGCAACGCCAAC-32
Tnfrsf1a F2/R2	mouse	52CTCCCTTCAACTTGATTTCCC-32	52CTTGGTCTCTGGTGGCTCTGT-32
Il-1²	mouse	5'-GAAATGCCACCTTTTGACAGTG-3'	5'-TGGATGCTCTCATCAGGACAG-3'
Tnf-±	mouse	5'-CAGGCGGTGCCTATGTCTC-3'	5'-CGATCACCCCGAAGTTCAGTAG -3'
Il-6	mouse	5'-CTGCAAGAGACTTCCATCCAG-3'	5'-AGTGGTATAGACAGGTCTGTTGG-3'
Ifi209	mouse	52ACCATCTCAAACTATGTTGGCTG-32	52TGGTTCGCCATCATTTACCTC-32
Gpr65	mouse	52CGCCACCTCGAACACTATTTG-32	52GCATAAAGATCCGATGTTGGCT-32
Marcksl1	mouse	52CCACCCGTGAACGGAACAG-32	52GGCCACTCAATTTGAAAGGCTT-32
Cxcr4	mouse	52GACTGGCATAGTCGGCAATG-32	52AGAAGGGGAGTGTGATGACAAA-32
Ifit3	mouse	52CCTACATAAAGCACCTAGATGGC-32	52ATGTGATAGTAGATCCAGGCGT-32
Treml2	mouse	52AGAACCGCCGGAACTTAGTTG-32	52TGATACGGACTACCTTGACCTTG-32
Bhlha15	mouse	52TGACCGCCACCATACTTACTA-32	52GCTGGTATAATTTAGGGCCTGG-32
Acvr1c	mouse	52ATGCTAACCAACGGGAAAGAG-32	52GGAAGGTGCAGTGTGATATTGT -32
NFºB	mouse	52ATGGCAGACGATGATCCCTAC-32	52CGGATCGAAATCCCCTCTGTT-32
Igkc	mouse	52GCTGTCCTGCTCATGTCCTT-32	52CACTGCCATCAATCTTCCACT-32
INF-±	mouse	52ATGGCTAGRCTCTGTGCTTTCCT-32	52AGGGGCTGTGTTTCTTCTCT-32
INF-³	mouse	52ATGAACGCTACACACTGCATC-32	52CCATCCTTTTGCCAGTTCCTC-32
Il-10	mouse	52GCTCTTACTGACTGGCATGAG-32	52CGCAGCTCTAGGAGCATGTG-32
IL-1²	Human	5'-ATGATGGCTTATTACAGTGGCAA-3'	5'-GTCGGAGATTCGTAGCTGGA-3'
IL-6	Human	5'-ACTCACCTCTTCAGAACGAATTG-3'	5'-CCATCTTTGGAAGGTTCAGGTTG-3'
IL-10	Human	5'-GACTTTAAGGGTTACCTGGGTTG-3'	5'-TCACATGCGCCTTGATGTCTG-3'

Supplementary Table S3 Different genes in bulk sequence					
gene	baseMean	logFC	lfcSE	P.Value	adj.P.Val
Col1a1	6317.28604	-1.31705988	0.24381244	3.8795E-10	1.7491E-07
D17H6S56E-5	4056.43707	1.520108216	0.19058499	4.849E-18	2.4048E-14
Apoe	4080.16103	-1.28945413	0.22117594	4.2405E-11	3.3205E-08
Gm10222	1205.14416	1.13905369	0.22402966	3.3394E-09	1.0766E-06
Lifr	1047.37554	1.099961982	0.13469706	7.7597E-18	2.8862E-14
Timp1	942.80568	-1.15534738	0.29159813	3.0899E-07	3.4053E-05
Bhlha15	935.135181	1.11532947	0.31399374	1.2843E-06	0.0001011
Tnfrsf1a	756.849025	-1.49052347	0.15206344	4.3153E-25	6.4204E-21
Postn	707.748229	-1.52724483	0.27507507	6.6092E-11	4.6824E-08
Mest	570.930139	1.17993652	0.34317474	1.4647E-06	0.0001135
Vill	534.031919	-1.00176674	0.16425304	1.6568E-11	1.3694E-08
Gpnmb	351.965185	-1.36323639	0.36698027	3.3086E-07	3.5414E-05
Tnc	315.297107	-1.2719731	0.51407201	8.1012E-06	0.00042291
Flrt2	317.177144	1.064446941	0.25354406	1.6068E-07	2.1344E-05
Slc15a3	299.827191	-1.05496763	0.25582043	2.15E-07	0.00002622
Kcnj15	302.552658	2.134219389	0.28587855	1.288E-17	3.8325E-14
Acta2	243.794612	-1.01765339	0.20188547	4.6499E-09	1.3836E-06
Cd163	230.731204	1.22402188	0.33087121	5.8646E-07	5.5223E-05
Pdlim7	224.276299	-1.08950474	0.21130582	2.5762E-09	9.1259E-07
Irf7	201.864784	-1.10123467	0.29152761	6.5634E-07	6.0278E-05
Mki67	197.863405	-1.01818818	0.20229792	4.8208E-09	1.3848E-06
Foxred2	182.778331	1.26196002	0.19479183	9.3997E-13	1.2714E-09
Cx3cr1	168.737764	-1.02603042	0.37231883	1.4231E-05	0.00064751
Marcksl1	170.609209	-1.0671913	0.24532374	8.9311E-08	1.3841E-05
Oas1a	161.633621	-1.0730355	0.25546004	1.573E-07	2.1084E-05
Obsl1	154.717798	-1.01480832	0.13815224	4.5945E-15	9.7653E-12
Lyve1	137.944385	1.090330141	0.23982931	3.9268E-08	7.3952E-06
Ifit3	128.991082	-1.05520236	0.32738153	3.9303E-06	0.00024186
Fcrl2	124.85014	-1.26998223	0.26099292	6.2413E-09	1.6291E-06
Cxcr4	124.753316	-1.12790784	0.26931472	1.4818E-07	2.0042E-05
Cilp	123.733511	-1.23989605	0.24554677	2.9761E-09	1.0297E-06
Dok3	123.489579	-1.11590245	0.28783774	4.5596E-07	4.5225E-05
Apol7a	129.821616	1.005235276	0.28440655	1.756E-06	0.00013087
Ccdc88b	117.468259	-1.04546591	0.4038028	1.9061E-05	0.000812
Pdk4	116.877551	1.364516753	0.2658052	1.2256E-09	4.9281E-07
Cyp4f18	106.557323	-1.13076584	0.34351201	2.5839E-06	0.00017881
Ifi209	103.855207	-1.00149169	0.26421257	7.5863E-07	6.6408E-05
Hnmt	98.1311187	1.034783317	0.17570535	5.6841E-11	4.2284E-08
Aplnr	85.6037639	-1.05459329	0.2106241	5.3943E-09	1.4809E-06
Arg2	90.5924936	1.073761664	0.30448569	1.549E-06	0.00011941
Aqp11	85.1737828	1.059683296	0.18160224	7.6751E-11	5.1905E-08
Il21r	79.3689193	-1.10483098	0.28193094	4.0591E-07	4.1364E-05
Slc6a4	80.9933627	1.272004467	0.23389698	3.8506E-10	1.7491E-07
Itm2a	81.5928075	-1.01508175	0.29089212	1.9834E-06	0.00014256
Thrb	78.598909	1.0899335	0.21813956	5.4746E-09	1.4809E-06

Gm49774	68.7804554	1.213822789	0.24088368	3.401E-09	1.0766E-06
Slc13a3	70.0035054	-1.0784382	0.74492299	0.00003248	0.00117576
Treml2	63.9283095	-1.55207138	0.36878991	2.8103E-08	5.8072E-06
Ttpa	68.9033452	1.293178786	0.30949983	9.342E-08	1.4265E-05
Oas3	59.1418205	-1.72955361	0.37033301	1.8709E-09	7.1372E-07
Ifit1	59.5858456	-1.05728278	0.34896347	6.5405E-06	0.00035277
Cpm	59.7235792	1.087625048	0.27755631	4.2165E-07	4.2387E-05
Gpr65	57.1978579	-1.07710615	0.25868555	1.7915E-07	2.2917E-05
AI467606	52.9043433	-1.12014488	0.34674569	3.1554E-06	0.0002059
Mfap4	52.8250903	-1.74770264	0.44221735	2.6204E-08	5.5694E-06
Iyd	52.9989445	1.724315682	0.25322915	1.1005E-14	2.0466E-11
Btnl9	51.5835006	1.189862989	0.30630546	3.6317E-07	3.8595E-05
Fbn2	45.2393833	-1.04771094	0.37853001	1.2781E-05	0.0005961
Il1b	46.2766884	-1.51737012	0.32938531	6.8522E-09	1.7279E-06
Fhl2	44.7459962	-1.2094789	0.2618632	2.2181E-08	4.9254E-06
Phf11b	44.9779185	-1.0396662	0.35318291	8.6182E-06	0.00043862
Ctse	43.668768	-1.64878339	0.41347096	3.8361E-08	7.3172E-06
Ckap2l	43.0188365	-1.116939	0.26616143	1.4712E-07	2.0042E-05
Kcnd3	41.6267283	1.013497836	0.39253897	2.2015E-05	0.00090732
Cdc20	39.5288978	-1.00438442	0.37941994	1.9764E-05	0.00083066
Rab44	37.4144469	-1.74849875	0.41575753	1.0863E-08	2.6068E-06
1810007D17Rik	40.6171698	4.637510125	0.55401599	8.5322E-19	6.3471E-15
Sult1a1	37.4276427	1.240353153	0.32464453	3.7956E-07	3.9871E-05
Kif5c	33.372246	1.029130631	0.33007594	5.6523E-06	0.00031241
Runx3	29.2529454	-1.09457459	0.44165698	1.9102E-05	0.000812
Angptl1	32.8093642	1.180555125	0.54246138	1.9042E-05	0.000812
C130050O18Rik	28.6491748	-1.0053604	0.43740397	3.9602E-05	0.00137023
Snhg11	29.7651819	2.016509216	0.45562892	1.0509E-09	4.343E-07
Slc4a11	26.3815802	-1.21954895	0.36535795	1.6744E-06	0.00012661
Lcat	27.9378685	1.296402823	0.3526637	4.8427E-07	4.7626E-05
Cmbl	26.5625988	1.129705807	0.37614029	5.4297E-06	0.00030593
Gm56577	23.2932569	1.458218793	0.33264094	2.2135E-08	4.9254E-06
Ccl21b	22.5335032	-2.07178706	0.63350303	3.0006E-08	6.0328E-06
Gm10591	22.5335032	-2.07178706	0.63350303	3.0006E-08	6.0328E-06
A530064D06Rik	21.0381313	-1.34054413	0.50980554	4.5224E-06	0.00026181
Siglecf	20.8614661	-1.09280437	0.54455366	3.6516E-05	0.00129354
Ccnb2	21.201582	-1.39285689	0.53429016	3.5845E-06	0.00022502
Tmprss13	20.8474163	1.277488132	0.34543962	4.8657E-07	4.7626E-05
Acvr1c	20.2138175	1.034191788	0.59217339	5.8382E-05	0.0018248
A1cf	20.0867376	1.367675265	0.48095936	2.7317E-06	0.00018473
Gm56738	18.4000685	1.097987438	0.54942391	3.5653E-05	0.00127189
Slc6a15	18.6833601	1.12179749	0.58122608	3.2182E-05	0.00116781
Phf11a	17.6756679	-1.31393857	0.51112231	5.6695E-06	0.00031241
Gabrp	16.1788796	-1.28327734	0.49697412	6.4958E-06	0.00035272
Cfap92	17.2818163	1.116831539	0.54082476	3.0285E-05	0.00112371
Gm15889	14.6452093	1.71182988	0.46327131	6.9404E-08	1.1224E-05
Rgs16	13.1706181	-1.04377719	0.4815436	0.00004017	0.00138147

Fam83d	9.25805102	-1.50300021	0.65751434	2.8766E-06	0.00019085
Thbs4	6.56021964	-1.16060003	1.3050715	1.3235E-05	0.00061154
